# Blood-brain barrier permeability increases with the differentiation of glioblastoma cells in vitro

**DOI:** 10.1186/s12987-024-00590-0

**Published:** 2024-11-01

**Authors:** Sabrina Digiovanni, Martina Lorenzati, Olga Teresa Bianciotto, Martina Godel, Simona Fontana, Muhlis Akman, Costanzo Costamagna, Pierre-Olivier Couraud, Annalisa Buffo, Joanna Kopecka, Chiara Riganti, Iris Chiara Salaroglio

**Affiliations:** 1 Department of Oncology, University of Torino, piazza Nizza 44, Torino, 10126 Italy; 2 Department of Neuroscience Rita Levi Montalcini, Institute Cavalieri Ottolenghi, Regione Gonzole 10, Orbassano, 10043 Italy; 3 Institute Cochin, 22 Rue Méchain, Paris, 75014 France; 4 Molecular Biotechnology Center “Guido Tarone”, University of Torino, piazza Nizza 44, Torino, 10126 Italy

**Keywords:** Blood-brain barrier, Glioblastoma multiforme, Glioblastoma stem cells, ATP Binding Cassette transporters, Tight junctions, Interleukin-6

## Abstract

**Background:**

Glioblastoma multiforme (GBM) is an aggressive tumor, difficult to treat pharmacologically because of the blood-brain barrier (BBB), which is rich in ATP-binding cassette (ABC) transporters and tight junction (TJ) proteins. The BBB is disrupted within GBM bulk, but it is competent in brain-adjacent-to-tumor areas, where eventual GBM foci can trigger tumor relapse. How GBM cells influence the permeability of BBB is poorly investigated.

**Methods:**

To clarify this point, we co-cultured human BBB models with 3 patient-derived GBM cells, after separating from each tumor the stem cell/neurosphere (SC/NS) and the differentiated/adherent cell (AC) components. Also, we set up cultures of BBB cells with the conditioned medium of NS or AC, enriched or depleted of IL-6. Extracellular cytokines were measured by protein arrays and ELISA. The intracellular signaling in BBB cells was measured by immunoblotting, in the presence of STAT3 pharmacological inhibitor or specific PROTAC. The competence of BBB was evaluated by permeability assays and TEER measurement.

**Results:**

The presence of GBM cells or their conditioned medium increased the permeability to doxorubicin, mitoxantrone and dextran-70, decreased TEER, down-regulated ABC transporters and TJ proteins at the transcriptional level. These effects were higher with AC or their medium than with NS. The secretome analysis identified IL-6 as significantly more produced by AC than by NS. Notably, AC-conditioned medium treated with an IL-6 neutralizing antibody reduced the BBB permeability to NS levels, while NS-conditioned medium enriched with IL-6 increased BBB permeability to AC levels. Mechanistically, IL-6 released by AC GBM cells activated STAT3 in BBB cells. In turn, STAT3 down-regulated ABC transporter and TJ expression, increased permeability and decreased TEER. The same effects were obtained in BBB cells treated with STA-21, a pharmacological inhibitor of STAT3, or with a PROTAC targeting STAT3.

**Conclusions:**

Our work demonstrates for the first time that the degree of GBM differentiation influences BBB permeability. The crosstalk between GBM cells that release IL-6 and BBB cells that respond by activating STAT3, controls the expression of ABC transporters and TJ proteins on BBB. These results may pave the way for novel therapeutic tools to tune BBB permeability and improve drug delivery to GBM.

**Supplementary Information:**

The online version contains supplementary material available at 10.1186/s12987-024-00590-0.

## Background

Glioblastoma multiforme (GBM) is the most common and lethal primary brain tumor in the adult population [1, 2]. Despite the advancements in the treatment options, including surgical resection, radiotherapy in combination with chemotherapy [3], and nanotechnologies-based approaches to improve drug delivery [4, 5], the prognosis of GBM patients is still poor, with a median survival of approximately 15 months. The main reasons are the highly invasive nature of GBM and the tumor chemoresistance [6].

Additionally, intratumor heterogeneity significantly contributes to GBM progression. Within the tumor bulk, cancer stem cells (CSCs), despite being in small proportion compared to differentiated cells, play a crucial role in tumor initiation, development, and recurrence [7, 8]. CSCs are characterized by a multidrug-resistant phenotype, because of the high levels of P-glycoprotein (Pgp), an ATP binding cassette (ABC) transporter that reduces the intracellular accumulation of drugs, including temozolomide [9, 10], the first-line therapy in GBM treatment. In addition, CSCs cause tumor relapse because of their intrinsic ability to disseminate in adjacent brain tissue [11].

The presence of the blood-brain barrier (BBB) further contributes to the intricate dynamics of drug resistance in GBM [12]. BBB is a physical and biological barrier for the delivery of chemotherapeutic drugs because it is rich in tight junctions (TJs) and adherent junctions that reduce paracellular fluxes and increase electrical resistance. Moreover, the ABC transporters abundant on the luminal surface, such as Pgp and breast cancer resistance protein (BCRP), efflux drugs back into the bloodstream [13, 14]. In GBM bulk the integrity of BBB is altered, as evidenced by contrast-enhanced magnetic resonance imaging [15, 16]. Conversely, BBB is intact in the so-called brain-adjacent-to-tumor (BAT) area, where isolated GBM cells are present. The differential integrity of BBB prevents the therapeutic agents from reaching GBM cells in the BAT area, leading to treatment failure and tumor relapse [17, 18].

Neuroinflammation, a pathophysiological condition associated with neurological disorders, such as Alzheimer’s disease, Parkinson’s disease [19, 20], multiple sclerosis, and ischemic stroke [21], but also with tumor progression and invasiveness, is linked to BBB breakdown [22]. GBM cells, together with astrocytes and microglia cells present within the tumor microenvironment (TME), release inflammatory mediators, such as interleukins (ILs) and chemokines that produce an immunosuppressive or inflammatory TME [23], alter BBB integrity and induce significant changes in permeability [24]. The molecular circuitries underlying these events at the GBM-BBB interface are poorly known.

In this work, we reported for the first time that the degree of differentiation or stemness of GBM cells differentially affects BBB permeability. We identified one druggable molecular circuitry, based on IL-6/Signal Transducer and Activator of Transcription 3 (STAT3) axis that could be exploited to increase drug permeability across BBB, improving the delivery of chemotherapeutic drugs to GBM.

## Methods

### Chemicals

Plasticware for cell cultures was obtained from Falcon (Becton Dickinson, Franklin Lakes, NJ). Electrophoresis reagents were obtained from Bio-Rad Laboratories (Hercules, CA). The protein content of cell lysates was assessed using the BCA kit from Merck-Sigma (St. Louis, MO). Unless specified otherwise, all reagents were purchased from Merck-Sigma.

### Cells

The immortalized hCMEC/D3 cells, primary human brain microvascular endothelial cells that retain BBB properties in vitro, were cultured as reported [25]. Cells were seeded at 50,000/cm^2^ density and were grown for 7 days up to confluence in Petri dishes and Transwell (0.4-µm diameter pore-size, Corning Life Sciences, Chorges, France). hCMEC/D3 cells were cultured in EndoGRO™ Basal Medium (Lonza, Basel Switzerland), 0.2% EndoGRO-LS Supplement, 5 ng/mL recombinant human (rh)EGF, 50 µg/mL ascorbic acid, 10 mM L-glutamine, 0.5 µM hydrocortisone hemisuccinate, 0.75 U/mL heparin sulphate, 2% fetal bovine serum (FBS; all from Lonza).

Human astrocytes (hAs) were differentiated from human Embryonic Stem Cells (hESCs – WA09, agreement no. 23W06046) as detailed in [26, 27]. Briefly, hESCs were differentiated into neural epithelium with dual-SMAD inhibitors 10 mM SB431542 (#1614/10, Tocris Bioscience, Bristol, UK) and 250 nM LDN193189 (#6053/10, Tocris), then into glial-committed cells by culture into N2 Medium: DMEM-F12 (#31330038) containing 1% GlutaMAX (#35050038), 1% MEM NEEA (#11140050), 0.1% 2-mercaptoethanol (#21985023; all purchased from ThermoFisher Scientific, Waltham, MA), 1 mM Smoothened Agonist (SAG; #6390/1, Tocris), 100 nM retinoic acid (#R2625; Sigma Merck) and 1% N2 supplement (#17502001; ThermoFisher Scientific). After mechanical dissociation, cells were cultured for 18 days in suspension as spheres in PDGF Medium: DMEM-F12 containing 1% GlutaMAX, 1% MEM NEEA, 0.1% 2-mercaptoethanol, 1% N2 supplement, 2% B27 (#17504044), 10 ng/mL PDGFa (#PHG0035), 10 ng/mL IGF-1 (#PHG0078), 5 ng/mL HGF (#PHG0254), 10 ng/mL NT3 (#PHC7036; all from Thermofisher Scientific), and 1% rh-insulin (#I3536), 60 ng/mL T3 (#T2877), 100 ng/mL biotin (#B4639), 1 mM cAMP (#A9501). Cells were then plated into 6-well plates coated with 0.1 mg/mL poly-L-ornithine (#P3655) and 10 mg/mL laminin (#L2020), in PDGF Medium. After 40 days, spheres and cells migrated out of the spheres were dissociated and sorted for CD49f positivity, using a PE-conjugated anti-CD49f antibody (#555736; BD Biosciences, New Jersey, USA; 1/50). CD49f^+^ sorted astrocytes were plated at a density of 20,000 cells/cm^2^ into poly-L-ornithine/laminin-coated 6-well plates for tri-culture BBB assembly, or into poly-L-ornithine/laminin coated µ-Slide 18 well ibiTreat (Ibidi, Gräfelfing, Germany) for morphological analysis. Human pericytes (hPEs; #C-12980, PromoCell, Heidelberg, Germany) were cultured in Pericyte Growth Medium 2 (#C-28041, PromoCell) as per the manufacturer’s instructions The hCMEC/D3-hAs-hPEs triculture was performed according to [28]. Briefly, 20,000 cells/cm^2^ hAs were seeded in pre-coated lower chambers of Transwell devices. The day after, 20,000 cells/cm^2^ hPEs were seeded in the reverse side of the insert, previously pre-coated. After 3 h, 50,000 cells/cm^2^ hCMEC/D3 were seeded in the insert. The triple culture was maintained for 7 days and then used for the experimental assays.

Primary human GBM cells (derived from 3 different patients, anonymized as #1, #2 and #3) were obtained from surgical samples of Neurosurgical Units, Universities of Torino and Novara, Italy, after written-informed consent (approval by the local Ethics Committee: #ORTO11WNST). Cells were used within passage 5 [10, 14]. Genetic background, clinical treatments and outcomes of patients are reported in the Supplementary Table S1. The histologic diagnosis of GBM (grade IV glioma) was performed according to World Health Organization (WHO) guidelines. Tumors were dissociated mechanically to obtain a single-cell suspension, and cells were cultured as differentiated/adherent cells (AC) or stem cells/neurospheres (NS) as previously described [9]. For AC, DMEM (#11965092, Thermofisher Scientific), 1% penicillin-streptomycin (#P4333) and 10% FBS were used. For NS, DMEM-F12 medium was supplemented with 1% v/v penicillin-streptomycin, 1 mM Hepes, 0.3 mg/mL glucose, 75 mg/mL NaHCO_3_, 2 mg/mL heparin, 2 mg/mL bovine serum albumin, 2 mM progesterone, 20 ng/mL EGF and 10 ng/mL b-FGF. In vitro clonogenicity, self-renewal and in vivo tumorigenicity of NS compared to AC are reported in the Supplementary Figure S1. Cell phenotypic characterization is detailed in the Supplementary Table S2. *Mycoplasma spp* contamination was assessed by PCR every 3 weeks; contaminated cells were discharged.

In a complementary experimental set, both AC and NS, dissociated as single cells, were seeded at the concentration of 3,000 cells/well into 24-well plates covered by rat collagen I (Invitrogen Life Technologies, Monza, Italy). After 72 h, the number of viable cells was counted under the microscope after Trypan blue (#T8154) staining, as per the manufacturer’s instructions.

In gene silencing experiments, AC cells were transfected with 0.5 µg of Human IL6 shRNA Plasmid (#abx959825, Abbexa Ltd, Cambridge, UK) or with a shRNA negative control, containing a scrambled sequence (#abx991273, Abbexa), using the JetPrime transfection reagents (Polyplus, Illkirch, France) as per manufacturer’s instructions. The efficacy of silencing was measured by quantifying the amount of IL-6 released in the supernatant after 24 and 48 h, as detailed below.

In co-culture systems, hCMEC/D3 cells were seeded in Transwell inserts; after 7 days, 3,000 GBM cells or 1.5 ml GBM-conditioned medium were added into the lower chamber. After 72 h of cell co-culture or medium co-incubation, the medium of the upper and lower chambers was replaced, and cells were used for the experimental assays.

### Permeability across the BBB monolayer and accumulation within GBM cells

The permeability to doxorubicin (DOX), mitoxantrone (MXR) and dextran 70-fluorescein isothiocyanate (DEXT) were used as parameters of Pgp activity (DOX), BCRP activity (MXR) and TJ integrity (DEXT) [25]. The assays were performed in hCMEC/D3 cells, co-cultured with GBM cells or GBM-conditioned medium for 72 h. In a second experimental set, rhIL-6 (#ab119444; Abcam, Cambridge, UK; 200 pg/ml) or neutralizing anti-IL6 antibody (NAb) (#ab6672, Abcam; 1/400, equivalent to 0.187 µg/mL) were added to NS-conditioned medium and AC-conditioned medium, respectively, 1 h before the assay. As control, hCMEC/D3 cells were grown for 72 h in DMEM containing IL-6 NAb and recombinant IL-6, without GBM cells or their conditioned medium. When indicated, the STAT3 inhibitor STA-21 (CAS 28882-53-3, Santa Cruz Biotechnology Inc., Santa Cruz, CA; 30 µM) or the proteolysis targeting chimera (PROTAC) against STAT3 SD-36 (#HY-129602, MedChemExpress, Monmouth Junction, NJ; 1 µM) were added in the Transwell insert containing hCMEC/D3 cells. At the end of the incubations, the culture medium was replaced in both chambers. 5 µM DOX, 10 µM MXR or 2 µM DEXT, were added to the upper chamber, alone or in the presence of the dual Pgp/BCRP inhibitor Elacridar (#HY-50879; MedChemExpress; 2 µM) in the lower chamber. After 3 h, the medium and the AC/NS cells in the lower chamber were collected. Cells were washed twice with PBS, sonicated and resuspended in 300 µL PBS. A 50 µL aliquot was used to measure the protein contents (BCA kit). The amounts of DOX, MXR and DEXT in the medium and lysates were measured fluorometrically, using a Multi-HX Synergy spectrofluorometer (Bio-Tek Instruments, Winooski, MT). Excitation and emission wavelengths were: 475 and 553 nm (DOX), 607 and 684 nm (MXR), 494 and 518 nm (DEXT). Fluorescence was converted in nmoles/cm^2^, using calibration curves set in each experiment. The permeability coefficients were calculated as reported [29]. The DOX, MXR and DEXT amount within GBM cells was expressed as nanomoles/mg cell proteins, according to the titration curves prepared in each experiment.

### Transendothelial electrical resistance (TEER)

hCMEC/D3 cells were seeded in Transwell inserts at 50,000 cells/cm^2^ and grew up to confluence for 7 days. After 72 h further of co-culture between BBB and GBM cells or their conditioned medium, TEER was measured using a Voltohmetro Millicell-ERS (Millipore, Bedford, MA), according to the manufacturer’s instructions. The mean TEER value of the plastic insert in the absence of cells was 26.73 ± 0.45 Ω cm^2^ (*n* = 8). This value was subtracted from each value obtained in the presence of the cells.

### Immunoblotting

Cells were rinsed with ice-cold lysis buffer (50 mM Tris, 10 mM EDTA, 1% v/v Triton-X100), supplemented with the protease inhibitor cocktail III (Merck-Sigma), sonicated and centrifuged at 13,000 × g for 10 min at 4 °C. 20 µg of proteins were subjected to SDS-PAGE and probed with the following antibodies: anti-claudin 3 (#34-1700, Invitrogen Life Technologies, 1/1000); anti-claudin 5 (clone 4C3C2, #35-250, Invitrogen Life Technologies, 1/1000); anti-occludin (clone OC-3F10, #33-1500, Invitrogen Life Technologies, 1/1000); anti-zonula occludens-1 (ZO-1) (clone 1A12, #33-9100, Invitrogen Life Technologies, 1/1000); anti-Pgp/ABCB1 (clone C219, #MA1-26528, Invitrogen Life Technologies, 1/1000), anti-BCRP/ABCG2 (clone BXP-21, #sc-58222, Santa Cruz Biotechnology Inc., 1/250), anti-STAT3 (#06-596, Millipore; 1/1000), anti-phospho(Tyr705)STAT3 (clone D3A7, #9145 Cell Signalling Technologies, Danvers, MA, 1/1000), anti-GADPH (clone 0411, #sc-47724, Santa Cruz Biotechnology Inc., 1/1000), anti-β-tubulin (clone D-10, #sc-5274, Santa Cruz Biotechnology Inc.; 1/500), followed by a peroxidase-conjugated secondary antibody (Bio-Rad Laboratories). Proteins were detected by ChemiDoc imaging system (Bio-Rad Laboratories). Band density was calculated with the ImageJ software.

### Quantitative RealTime PCR (qRT-PCR)

RNA was retrotranscribed using the QuantiTect Reverse Transcription Kit (Bio-Rad Laboratories). qRT-PCR was carried out using IQ SYBR Green Supermix (Bio-Rad Laboratories), according to the manufacturer’s instructions. The same cDNA was used to quantify the genes of interest and the housekeeping gene (*S14*). Primer sequences were designed with the Primer3 software. Gene expression was calculated using the Gene Expression Quantitation software (Bio-Rad Laboratories).

### Cytokine array

GBM medium was obtained by 3,000 cells grown for 72 h in a 6-well plate. The medium was filtered through a 0.22 μm filter and stored at − 80 °C until the use. The secretion of cytokines in the GBM medium was analysed using a Human Cytokine Antibody Array (#Ab133998; Abcam), according to the manufacturer’s instructions. Chemiluminescent detection was performed with a ChemiDoc imaging system (Bio-Rad Laboratories).

### ELISA

The conditioned medium from AC and NS, seeded at the same density, were collected after 72 h and centrifuged at 1,000 × g for 10 min to remove debris. The levels of chemokine (C-C motif) ligand 2/monocyte chemoattractant protein 1 (CCL2/MCP-1), chemokine (C-X-C motif) ligand 8/interleukin-8 (CXCL8/IL-8), tumor necrosis factor-α (TNF-α) and IL-6 were measured by ELISA, using the Human MCP-1 (CCL2) Standard ABTS ELISA Kit (#900-K31, ImmunoTools, Friesoythe Germany), the Human IL-8 Elisa Kit (#31670089, ImmunoTools), the TNF-α ELISA kit (#31673019, ImmunoTools) and the Human IL-6 ELISA Kit High Sensitivity (#ab4604, Abcam). The absorbance was measured using a Multi-HX Synergy spectrofluorometer (Bio-Tek Instruments). A standard curve was used to calculate the levels of chemokines and cytokines. Results were expressed in picograms/mL.

### Statistical analysis

All data in text and figures are provided as means ± SD. The results were analysed by one-way analysis of variance (ANOVA) and Tukey’s test using GraphPad Prism 9.0 (GraphPad Software, San Diego, CA). *p* < 0.05 was considered significant.

## Results

### Glioblastoma multiforme cells and medium increase blood-brain barrier permeability by down-regulating ABC transporter and tight junction proteins

**Fig. 1:**
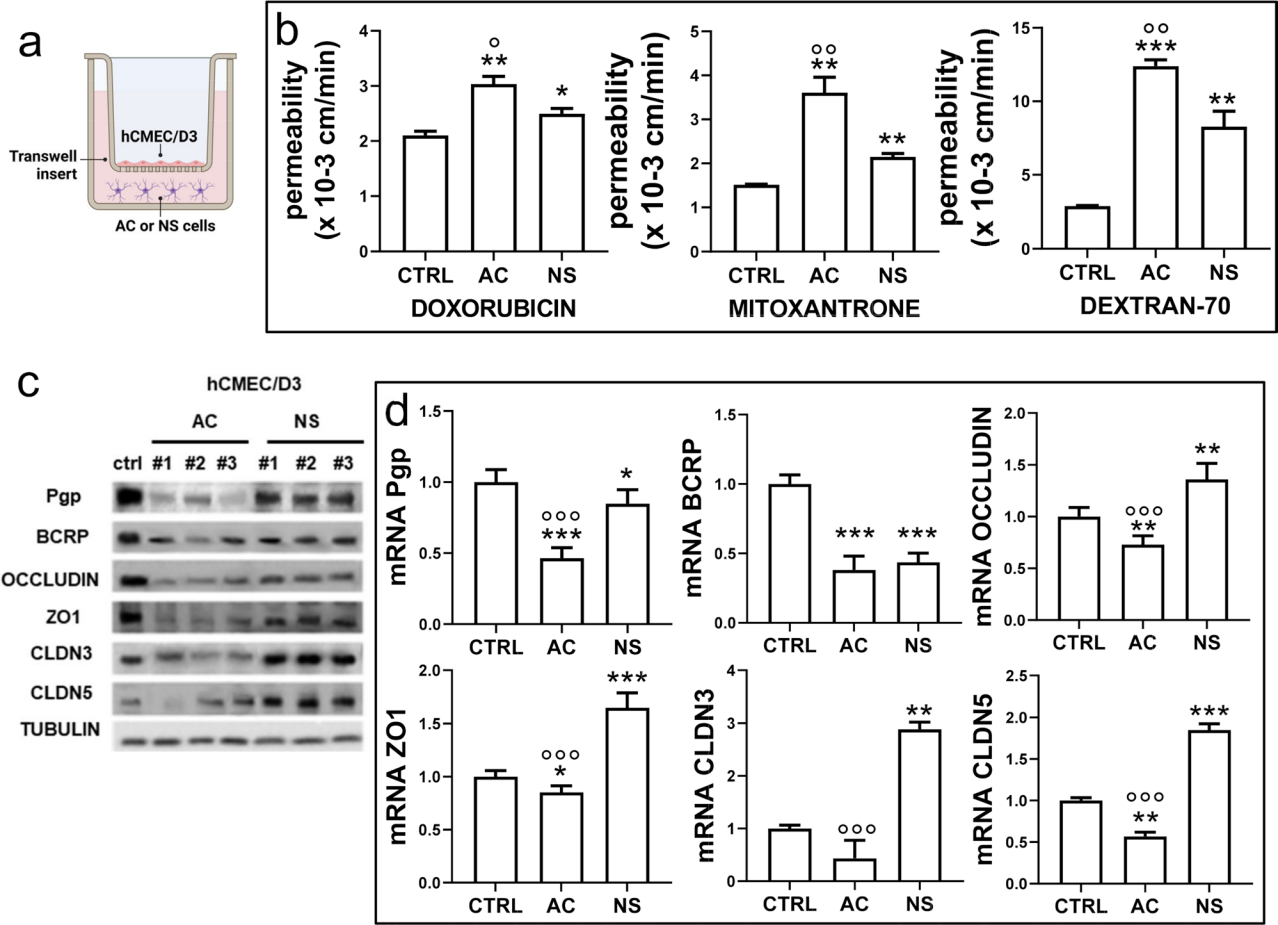
Glioblastoma cells affect BBB permeability, decreasing ABC transporters and tight junction proteins. **a**. Schematic representation of blood-brain barrier (BBB)-glioblastoma (GBM) co-cultures. Human BBB hCMEC/D3 cells were grown up to confluence in Transwell inserts. After 7 days, GBM cells derived from patients #1, #2, #3, as differentiated/adherent cells (AC) or stem cell/neurospheres (NS), were added in the lower chamber for 72 h. Then, the medium in the upper and lower chambers was replaced and the co-cultures were used for the experimental assays. A Transwell containing BBB cells only, grown for 7 days, was used as control (CTRL). **b**. Permeability assays. 5 µM doxorubicin, 10 µM mitoxantrone or 2 µM dextran 70-fluorescein isothiocyanate were added for 3 h. The compounds recovered from the lower chamber were measured fluorometrically. Data were the means ± SD of the results obtained from patients #1, #2, #3, whose disaggregated data are reported in Supplementary Figure S2a. *p < 0.05, **p < 0.01, ***p < 0.001: AC/NS vs. CTRL; °*p* < 0.05, °°*p* < 0.01: AC vs. NS. **c**. Immunoblotting of ABC transporters and tight junction (TJ) proteins in BBB cells. The expression of tubulin was used as control of equal protein loading. The figure is representative of one out of three experiments with similar results. ZO-1: zonula occludens 1; CLDN3: claudin-3; CLDN5: claudin-5. **d.** qRT-PCR of ABC transporters and TJ genes in BBB cells. Data were the means ± SD of the results obtained from patients #1, #2, #3, whose disaggregated data are reported in Supplementary Figure S2c. The expression level of CTRL BBB cells was considered “1” and used as the reference for all the other experimental conditions. *p < 0.05, **p < 0.01, ***p < 0.001: AC/NS vs. CTRL; °°°*p* < 0.001: AC vs. NS

Since BBB is disrupted in the bulk of GBM [16], where different tumor populations co-exist, we investigated whether differentiated (AC) or stem cells (NS) GBM cells could influence the permeability of BBB and the expression of ABC transporters and TJ proteins. We set up BBB-GBM co-culture models by seeding the hCMEC/D3 cells monolayer in the upper chamber of Transwell inserts and primary GBM cells derived from patients, cultured as AC or NS in the lower chamber (Fig. 1a). For the sake of simplicity, Fig. 1b (permeability assays) and 1d (qRT-PCR assays) show the means ± SD of the results of 3 independent experiments performed in each patient-derived sample. Since GBM has high interpatient variability, the results of each patient are shown separately in the Supplementary Figure S2. Immunoblot experiments (Fig. 1c) and densitometric quantifications are reported as data for each patient-derived sample in both Fig. 1 and Supplementary Figure S2.

After 72 h of co-culture with GBM cells the apical-to-basolateral permeability of DOX (a substrate of Pgp), MXR (a substrate of BCRP) and DEXT (transported when TJs are impaired) was increased compared to hCMEC/D3 monolayer alone (Fig. 1b, Supplementary Figure S2a). Notably, these effects were greater and more consistent in the presence of AC than in the presence of NS (Fig. 1b, Supplementary Figure S2a). Indeed, in the case of ABC transporters, Pgp and BCRP were decreased in BBB co-cultured with AC GBM cells compared to BBB alone (Fig. 1c), with the same trend in each patient (Supplementary Figure S2b). The decrease was less pronounced in BBB co-cultured with NS and showed higher interpatient variability (Fig. 1c, Supplementary Figure S2b). In the case of TJ proteins, the scenario is more variegated: occludin and ZO-1 were equally decreased in AC GBM/BBB and NS GBM/BBB co-cultures, claudin-3 and claudin-5 were unchanged and increased, respectively, in AC GBM/BBB co-cultures, and both increased in NS GBM/BBB co-cultures (Fig. 1c, Supplementary Figure S2b). Also, claudin-5 displayed the highest variability between the three patient-derived AC/GBM co-cultures (Supplementary Figure S2b). The co-culture with AC decreased the mRNA levels of Pgp, BCRP, ZO-1, occludin and claudin-5, despite the interpatient variability evident in claudin-5. A similar but not significant decrease was observed for claudin-3 (Fig. 1d, Supplementary Figure S2c). NS cells produced minor and variable changes: Pgp was decreased significantly in #2 and #3 NS/BBB co-culture and slightly reduced in #1 NS/BBB co-cultures; BCRP was significantly decreased in all co-cultures (Fig. 1d, Supplementary Figure S2c). TJ mRNAs were increased in NS/BBB co-cultures (Fig. 1d), at different extents between each patient (Supplementary Figure S2c). TEER values were decreased in BBB monolayer co-cultured with AC and NS, with a greater decrease induced by AC (Supplementary Figure S3).

To verify if the changes in DOX and MXR transport were due to the specific decrease in Pgp/BCRP activity rather than to a general disruption of BBB integrity, we added the dual Pgp/BCRP inhibitor Elacridar in the lower chamber of the co-cultures between BBB and NS #2. We detected higher permeability of DOX and MXR across hCMEC/D3 monolayer in co-cultures treated with Elacridar, compared to untreated co-cultures (Supplementary Figure S4a). DOX and MXR were also more accumulated within NS, rich in Pgp and BCRP [9], in co-cultures exposed to Elacridar (Supplementary Figure S4b), reaching values like the co-cultures of BBB and AC. As expected, no changes were observed for DEXT, since it is not a substrate of Pgp or BCRP. These results indicate that Elacridar may increase BBB permeability and intratumor retention of both DOX and MXR by inhibiting Pgp and BCRP present in both endothelial and GBM cells, without altering transport processes dependent on TJ integrity.

**Fig. 2:**
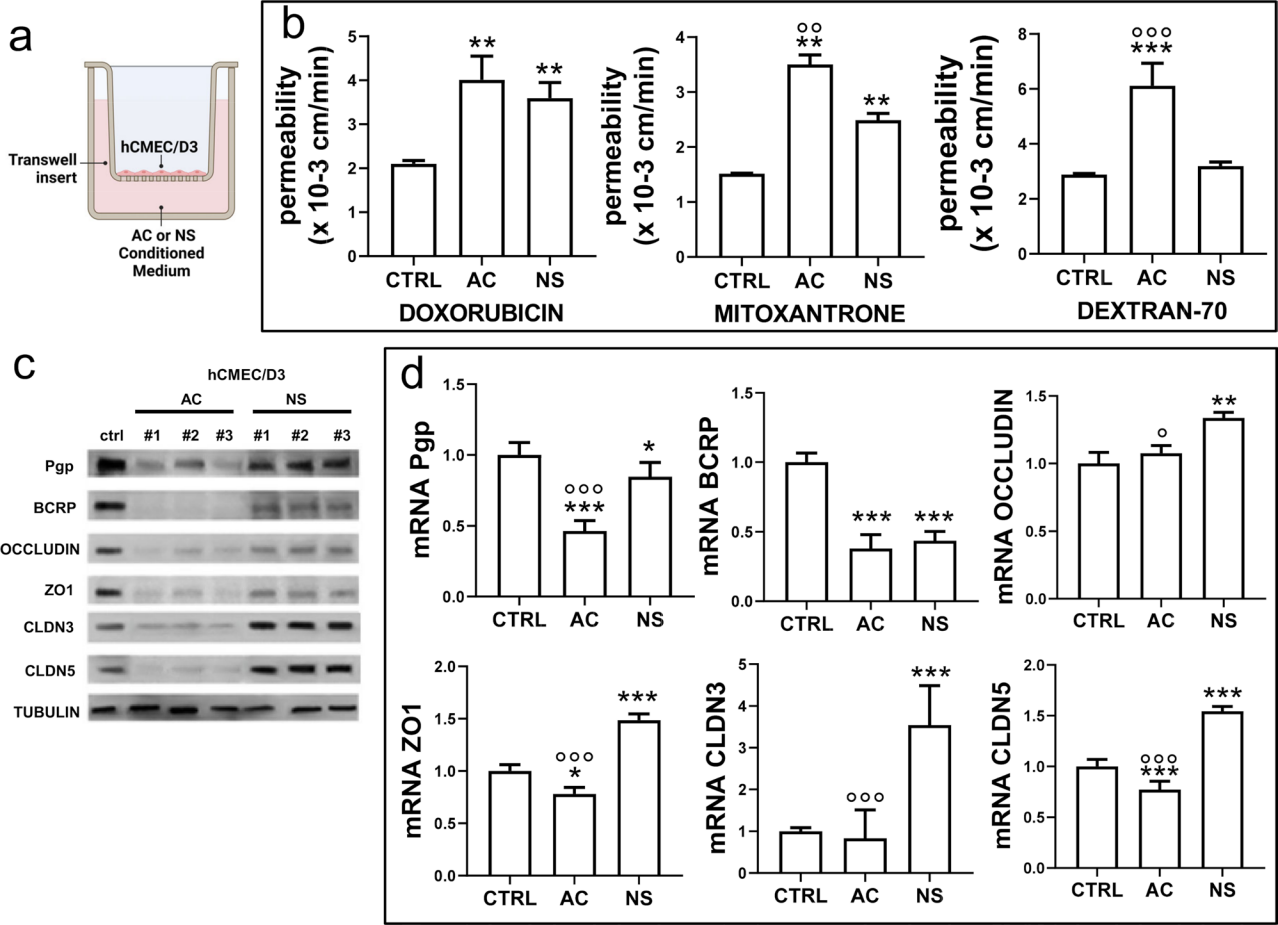
The conditioned medium of glioblastoma changes BBB permeability. **a**. Schematic representation of blood-brain barrier (BBB)-conditioned medium cultures. Human BBB hCMEC/D3 cells were grown up to confluence in Transwell inserts. After 7 days, the conditioned medium derived from 5-day culture of GBM cells of patients #1, #2, #3, either from differentiated/adherent cells (AC) or stem cell/neurospheres (NS), was added in the lower chamber for 72 h. Then the medium in the upper and lower chamber was replaced, and the cultures were used for the experimental assays. A Transwell containing BBB cells only, grown for 7 days, was used as control (CTRL). **b**. Permeability assays. 5 µM doxorubicin, 10 µM mitoxantrone or 2 µM dextran 70-fluorescein isothiocyanate were added for 3 h. The compounds recovered from the lower chamber were measured fluorometrically. Data were the means ± SD of the results obtained from patients #1, #2, #3, whose disaggregated data are reported in Supplementary Figure S5a. **p < 0.01, ***p < 0.001: AC/NS vs. CTRL; °°*p* < 0.01, °°°*p* < 0.001: AC vs. NS. **c**. Immunoblotting of ABC transporters and tight junction (TJ) proteins in BBB cells. The expression of tubulin was used as control of equal protein loading. The figure is representative of one out of three experiments with similar results. ZO1: zonula occludens 1; CLDN3: claudin-3; CLDN5: claudin-5. **d**. qRT-PCR of ABC transporters and TJ genes in BBB cells. Data were the means ± SD of the results obtained from patients #1, #2, #3, whose disaggregated data are reported in Supplementary Figure S5c. The expression level of CTRL BBB cells was considered “1” and used as the reference for all the other experimental conditions. *p < 0.05, **p < 0.01, ***p < 0.001: AC/NS vs. CTRL; °*p* < 0.05, °°°*p* < 0.001: AC vs. NS

Interestingly, the conditioned medium obtained from a 5 day-culture of NS or AC, added for 72 h in the lower chamber of Transwell devices, with hCMEC/D3 monolayer in the inserts (Fig. 2a), increased the permeability to DOX, MXR and DEXT respect to hCMEC/D3 monolayer alone (Fig. 2b: means ± SD of 3 patient-derived samples, Supplementary Figure S5a: disaggregated data from each patient). Again, AC-conditioned medium induced a higher increase in the permeability than NS-conditioned medium (Fig. 2b, Supplementary Figure S5a). The conditioned medium also recapitulated the changes in ABC transporter proteins (Fig. 2c, Supplementary Figure S5b) and mRNAs (Fig. 2d: means ± SD of the 3 patient-derived samples, Supplementary Figure S5c: disaggregated data from each single patient). In the case of AC-derived conditioned medium, Pgp and BCRP were significantly decreased at protein and mRNA levels (Fig. 2c-d, Supplementary Figure S5b-c), except for the mRNA derived from the treatment with the medium of patient #1. NS-derived conditioned medium produced a mild decrease in Pgp and a clearer decrease in BCRP protein and mRNA (Fig. 2c-d) with higher interpatient variability (Supplementary Figure S5b-c). TJ proteins were all homogeneously decreased by AC-conditioned medium (Fig. 2c, Supplementary Figure S5b), slightly (occludin, ZO-1) or strongly (claudin-3, claudin-5) increased by NS-conditioned medium following this rank order in terms of increase: patient #1 < patient #2 < patient #3 (Supplementary Figure S5b). The same trend – i.e., a modest increase in occludin and ZO-1, a stronger increase in claudin-3 and claudin-5 (Fig. 2d, Supplementary Figure S5c) – was observed in the mRNA of the TJ genes.

This experimental set suggested that soluble factors differentially released by AC and NS may produce the permeability changes observed in BBB, modulating ABC transporter and TJ levels. The changes produced by the conditioned medium on ABC transporters were in line with those produced by the co-cultures of GBM and BBB cells, particularly in the case of AC-derived medium. As far as TJ proteins are concerned, the results obtained with the conditioned medium do not fully recapitulate those obtained in the GBM/BBB co-cultures, because of the higher variability detected between the patients.

**Fig. 3:**
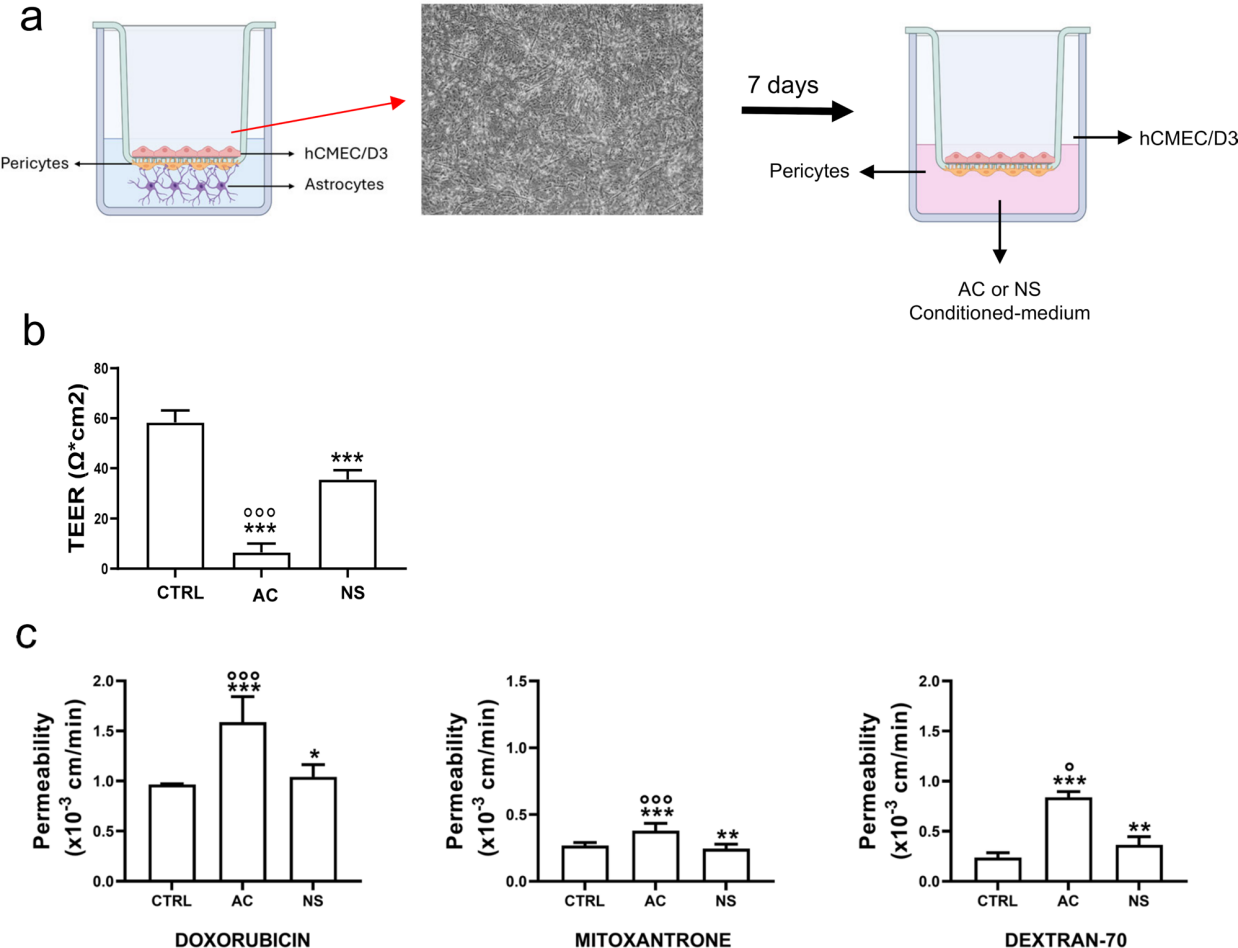
Effects of glioblastoma multiforme medium on BBB model made by endothelial cells, astrocytes and pericytes. **a**. A triculture-based model of the blood-brain barrier (BBB) was performed by seeding human hCMEC/D3 cells in the Transwell insert, human pericytes on the reverse insert and human astrocytes in the lower chamber (left panel). After 7 days, the conditioned medium derived from 5-day culture of GBM cells of patient #2, either from differentiated/adherent cells (AC) or stem cell/neurospheres (NS), was added in the lower chamber for 72 h (right panel). Then the medium in the upper and lower chambers was replaced and the cultures were used for the experimental assays. A Transwell containing BBB cells only, grown for 7 days, was used as control (CTRL). **b**. TEER values of BBB culture. Data are presented as mean ± SD (*n* = 3 independent experiments; each experimental point was performed in technical duplicates). ***p < 0.0001: AC/NS vs. CTRL; °°°*p* < 0.001: AC vs. NS. **c**. Permeability assays. 5 µM doxorubicin, 10 µM mitoxantrone or 2 µM dextran 70-fluorescein isothiocyanate were added for 3 h. The compounds recovered from the lower chamber were measured fluorometrically. Data are presented as means ± SD (*n* = 3 independent experiments; each experimental point was performed in technical duplicates). *p < 0.05, **p < 0.01, ***p < 0.001: AC/NS vs. CTRL; °*p* < 0.05, °°°*p* < 0.001: AC vs. NS

To consolidate our data in a model closer to the physiological BBB, we set up a triculture containing hCMEC/D3 cells co-cultured with hPEs and hAs (Fig. 3a, Supplementary Figure S6a). In the triculture-based BBB model, TEER value was higher (Supplementary Figure S6b) and permeability to DEXT was lower (Supplementary Figure S6c) than in monoculture-based model, containing the hCMEC/D3 monolayer only. The permeability to DOX was slightly decreased, while the permeability of MXR was unchanged (Supplementary Figure S6c). Next, we added the conditioned medium from AC and NS in the lower chamber of the triculture-based BBB model for 72 h. Notably, we obtained the same results as the monoculture-based BBB model: the GBM-conditioned medium decreased TEER values (Fig. 3b) and increased the permeability to DOX, MXR and DEXT (Fig. 3c). The effects were greater with the AC-derived medium (Fig. 3b-c).

## IL-6 was produced by differentiated glioblastoma cells more than by the stem cell counterpart

**Fig. 4:**
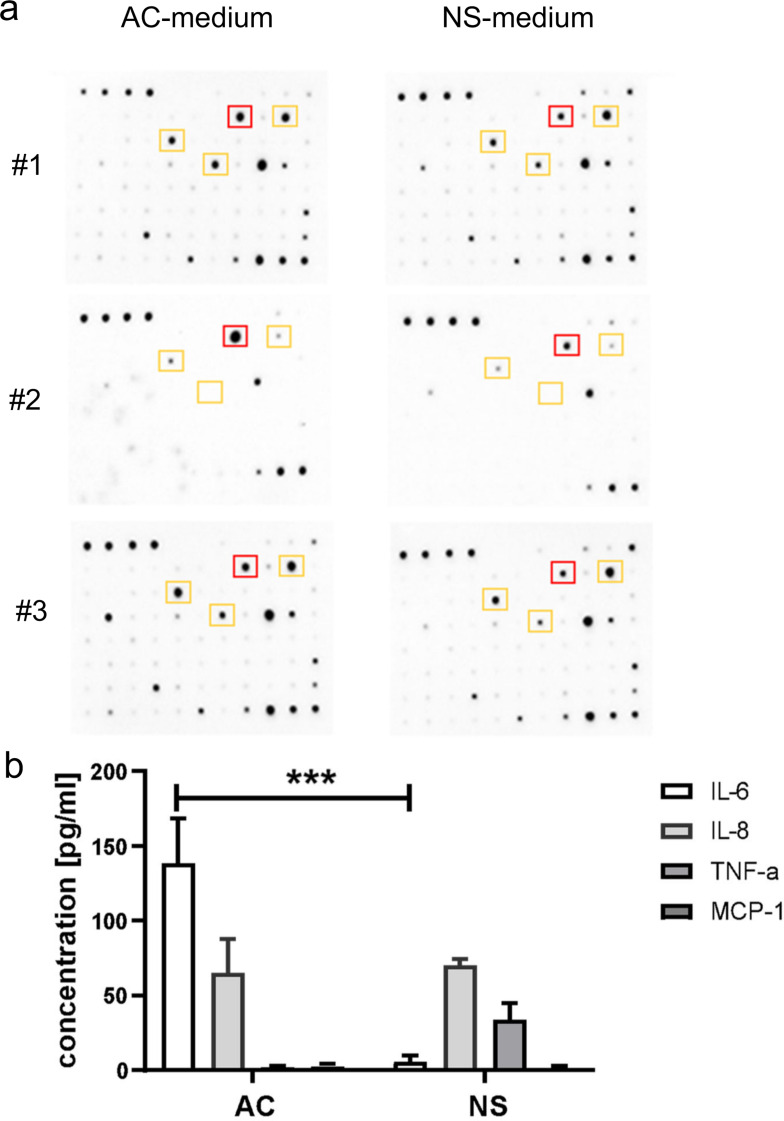
Identification of cytokines released by patient-derived glioblastoma cells. **a**. Cytokine levels in the medium of differentiated/adherent cells (AC) or stem cell/neurospheres (NS) of GBM cells derived from patients #1, #2 and #3, evaluated by a chemiluminescence-based array. The figure is representative of one out of three experiments with similar results. Squares identify the differentially expressed cytokines common to all patients. Red: IL-6; yellow: IL-8, TNF-α, MCP-1. **b**. ELISA detection of IL-6, IL-8, TNF-α, MCP-1 in AC- or NS-derived medium. Data were presented as mean ± SD (*n* = 3 independent experiments; each experimental point was performed in technical duplicates). ***p < 0.001: AC vs. NS

Considering the impact that soluble inflammatory mediators have on BBB permeability [30], we analyzed the secretome of AC and NS cells, and measured the amounts of cytokines and chemokines present. By screening the AC and NS media for 60 cytokines/chemokines with a specific array (Supplementary Figure S7), IL-6, IL-8, TNF-α and MCP-1 resulted higher in AC-medium compared to NS-medium in each patient (Fig. 4a). ELISA indicated IL-6 as the most differentially produced between AC and NS among these four cytokines (Fig. 4b). Since NS grow as spheres and proliferate more than AC cells [9] but may have inner cells more prone to apoptosis, we verified that the differences in IL-6 were not due to different proliferation or culture conditions of NS and AC. To this aim, we forced NS cells, dissociated as single cells, to grow on a collagen layer, in adherent conditions as AC. After 72 h, the number of viable AC and NS was the same for all GBM AC and NS samples (Supplementary Figure S8a). Additionally, we confirmed that AC secreted significantly more IL-6 than NS (Supplementary Figure S8b). These results suggested that the differential production of IL-6 was not a byproduct, caused by different proliferation and/or apoptosis rate between NS and AC.

## IL-6 released by glioblastoma cells is sufficient to increase the blood-brain barrier permeability

**Fig. 5:**
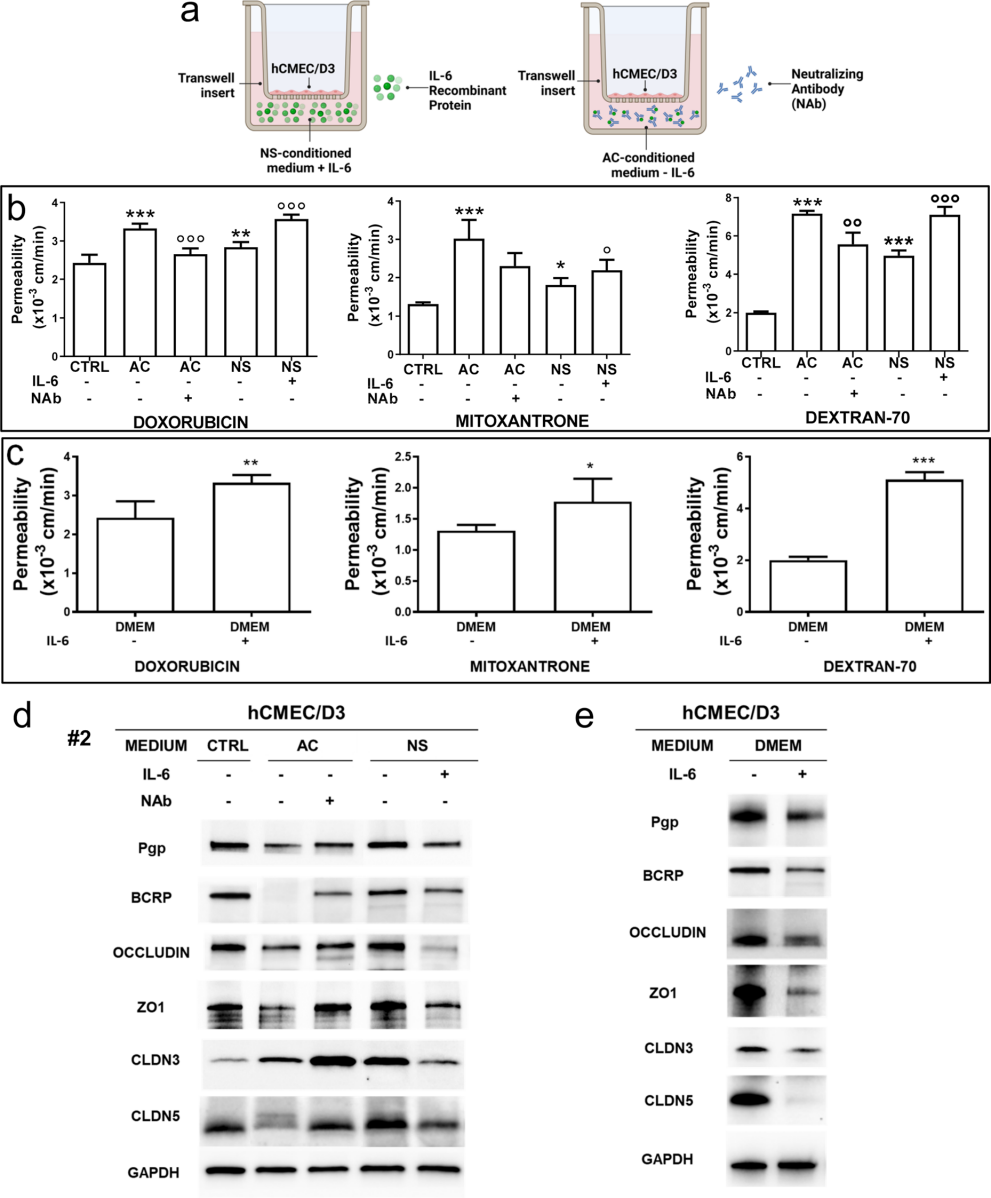
IL-6 released by glioblastoma increases BBB permeability by decreasing ABC transporter and tight junction proteins. **a**. Experimental design. Human blood-brain barrier (BBB) hCMEC/D3 cells were grown up to confluence in Transwell inserts. After 7 days, the conditioned medium derived from a 5-day culture of GBM cells of patient #2, as medium of differentiated/adherent cells (AC) or stem cell/neurospheres (NS), was added in the lower chamber for 72 h. rhIL-6 (200 pg/mL) was added in NS-conditioned medium, an anti-IL6 neutralizing antibody (Nab, 1/400) was added in AC-conditioned medium. Then the medium in the upper and lower chamber was replaced and the cultures were used for the experimental assays. A Transwell containing BBB cells only, grown for 7 days, was used as control (CTRL). **b**. Permeability assay. 5 µM doxorubicin, 10 µM mitoxantrone or 2 µM dextran 70-fluorescein isothiocyanate were added for 3 h. The compounds recovered from the lower chamber were measured fluorometrically. Data were presented as a mean ± SD (*n* = 3 independent experiments; each experimental point was performed in technical duplicates). *p < 0.05, **p < 0.01, ***p < 0.001: AC/NS vs. CTRL; °*p* < 0.05, °°*p* < 0.01, °°°*p* < 0.001: Nab-treated-AC vs. AC, IL-6-treated NS vs. NS. **c**. hCMEC/D3 cells were grown for 7 days up to confluence in Transwell insert, then incubated for 72 h with DMEM medium (used as control) or DMEM medium supplemented with rhIL-6 (200 pg/mL) in the lower chamber. The medium in both chambers was changed and a permeability assay was carried out as reported in **b**. Data were presented as mean ± SD (*n* = 3 independent experiments; each experimental point was performed in technical duplicates). *p < 0.05, **p < 0.01, ***p < 0.001: DMEM + IL-6 vs. DMEM. **d-e**. Immunoblotting of ABC transporter and TJ proteins in BBB cells, treated as reported in **a** and **c**. The expression of GAPDH was used as control of equal protein loading. The figure is representative of one out of three experiments with similar results. ZO1: zonula occludens 1; CLDN3: claudin-3; CLDN5: claudin-5

To clarify if IL-6 was sufficient to modulate BBB permeability, we designed two complementary culture sets, exposing for 72 h the hCMEC/D3 monolayer to (1) the NS-conditioned medium supplemented with 200 pg/mL rhIL-6 (corresponding to the concentration of AC medium; Figs. 4b and 5a: left panel); (2) the AC-conditioned medium depleted of IL-6, by using an anti-IL6 NAb that brings the concentration of the free cytokine levels to the very low level of NS medium (Figs. 4b, 5a: right panel). Interestingly, the permeability to DOX, MXR and DEXT was decreased in BBB monolayer co-cultured with IL-6-depleted AC-conditioned medium compared to AC-conditioned medium (Fig. 5b), while the mRNAs of ABC transporter and TJ proteins was increased (Supplementary Figure S9). Conversely, IL-6-supplemented NS-conditioned medium increased the permeability to DOX, MXR and DEXT compared to BBB monolayer co-cultured with NS-conditioned medium (Fig. 5b), and decreased the mRNAs of ABC transporter and TJ proteins (Supplementary Figure S9). There were no significant differences between AC-conditioned medium versus IL-6-supplemented NS-conditioned medium, nor between NS-conditioned medium versus IL-6-depleted AC-conditioned medium. In line with the results on BBB permeability, IL-6-depleted AC-conditioned medium increased and IL-6-supplemented NS-conditioned medium decreased TEER compared to AC- or NS-conditioned medium, respectively (Supplementary Figure S10a).

As proof of concept that IL-6 alone was sufficient to change BBB permeability, we cultured BBB monolayer with DMEM medium, supplemented with rhIL-6 at the same concentration found in AC-conditioned medium: the addition of rhIL-6 significantly increased the permeability to DOX, MXR and DEXT compared to parental DMEM (Fig. 5c). Similarly, IL-6 added to the DMEM medium decreased TEER to values comparable to the AC-conditioned medium (Supplementary Figure S10b).

Adding or depleting IL-6 also modulated ABC transporters and TJ proteins in BBB cells. IL-6-depleted AC-conditioned medium increased Pgp, BCRP, occludin, ZO-1, claudin-3 and claudin-5 compared to AC-conditioned medium, while IL-6-supplemented NS-conditioned medium decreased ABC transporters and TJ proteins compared to NS-conditioned medium, at protein and mRNA levels (Fig. 5d, Supplementary Figure S11a). Again, rhIL-6 in DMEM medium decreased Pgp, BCRP, occludin, ZO-1, claudin-3 and claudin-5 (Fig. 5e, Supplementary Figure S11b).

As an additional control, IL-6 was silenced in AC cells derived from patients 1#, 2# and #3 (Supplementary Figure S12a). The conditioned medium derived from AC or shIL-6 AC was added in the lower chamber of Transwell inserts containing a monoculture-based model (hCMEC/D3 only; Supplementary Figure S12b) or a triculture-based model (hCMEC/D3, hPEs and hAs; Supplementary Figure S12c). The permeability to DOX, MXR and DEXT was measured as endpoint assay. In both models, the silencing of IL-6 in AC abrogated the increase in permeability induced by AC medium, for all the substrates (Supplementary Figure S12b-c).

Overall, these data suggest that IL-6 released by GBM cells, particularly by the differentiated component, strongly increases BBB permeability.

### IL-6/STAT3 axis controls the expression of ABC transporter and tight junction proteins at the blood-brain barrier/glioblastoma interface

**Fig. 6:**
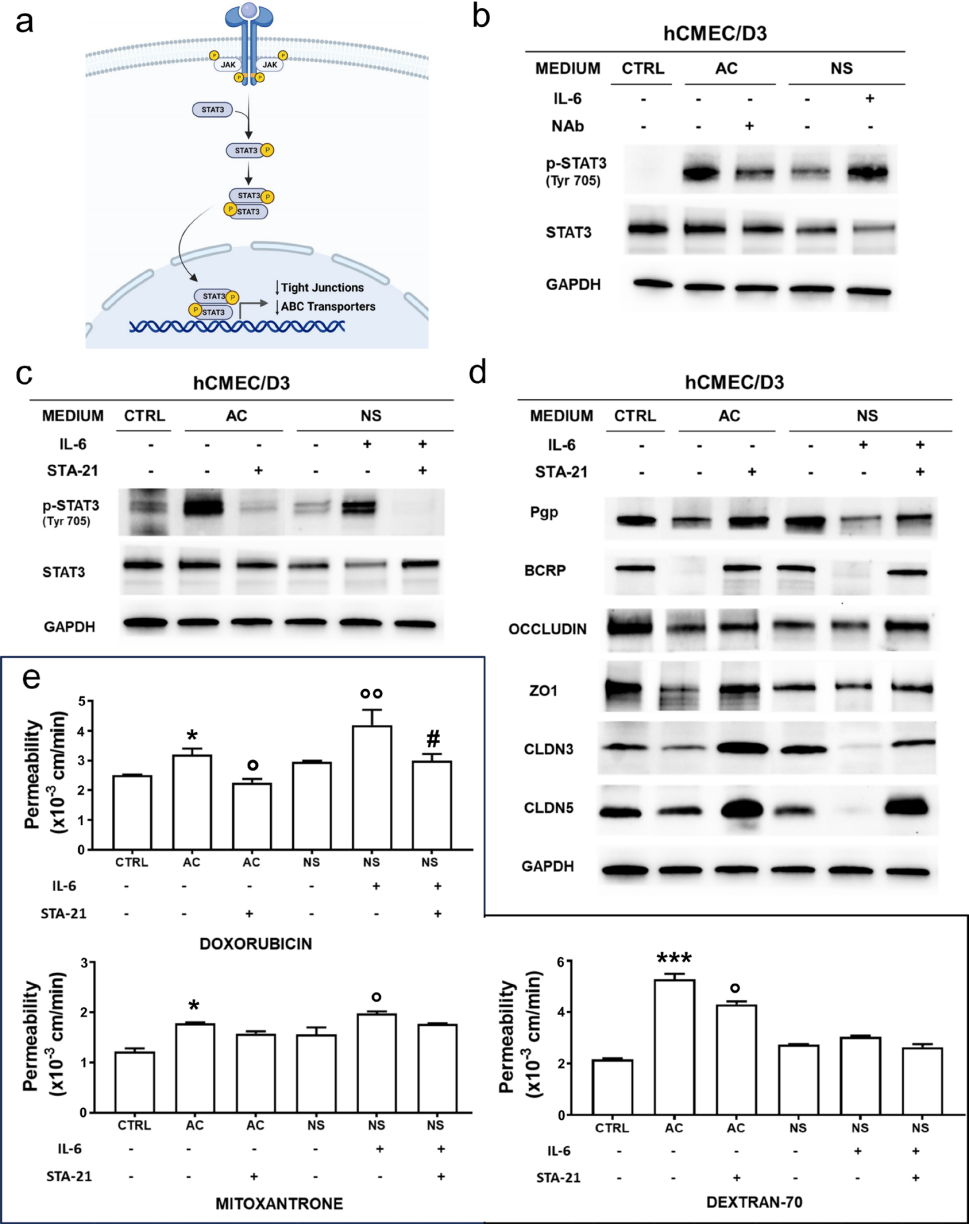
IL-6 modulates BBB permeability via STAT3 activation in blood-brain barrier cells. **(a)** Schematic representation of IL-6/STAT3 signalling. **(b)** hCMEC/D3 cells, grown for 7 days up to confluence in Transwell insert, were left untreated (CTRL) or incubated in the lower chamber with the conditioned medium derived from 5-day culture of GBM cells of patient #2, as medium from differentiated/adherent cells (AC), alone or containing an anti-IL-6 neutralizing antibody (Nab, 1/400), or medium from neurospheres (NS), alone or containing rhIL-6 (200 pg/mL). Immunoblot of STAT3 and phospho(Tyr705)STAT3. The expression of GAPDH was used as control of equal protein loading. The figure is representative of one out of three experiments with similar results. **(c)** BBB cells were grown as in **b**. When indicated the inhibitor of STAT3, STA-21 (30 µM) was co-incubated. Immunoblot of STAT3 and phospho(Tyr705)STAT3. The expression of GAPDH was used as control of equal protein loading. The figure is representative of one out of three experiments with similar results. **(d)** Immunoblotting of the indicated proteins in BBB cells, incubated as in **c**. The expression of GAPDH was used as control of equal protein loading. The figure is representative of one out of three experiments with similar results. ZO1: zonula occludens 1; CLDN3: claudin-3; CLDN5: claudin-5. **(e)** Permeability assay on BBB cells treated as in **c**. 5 µM doxorubicin, 10 µM mitoxantrone or 2 µM dextran 70-fluorescein isothiocyanate were added for 3 h. The compounds recovered from the lower chamber were measured fluorometrically, in duplicates. Data were presented as mean ± SD (*n* = 3 independent experiments). *p < 0.05, ***p < 0.001: AC/NS vs. CTRL; °*p* < 0.05, °°*p* < 0.01: STA-21-treated AC vs. AC, IL-6-treated NS vs. NS; ^#^*p* < 0.05; STA-21 + IL-6 treated NS vs. IL-6 treated NS

Next, we analyzed the IL-6-dependent signalling in BBB cells. We focused on the IL-6/STAT3 axis, which is activated by IL-6 in GBM [31, 32] and BBB [33, 34] (Fig. 6a). The phosphorylated active form of STAT3 was not detected in the BBB monolayer, but the GBM-conditioned medium, particularly the AC-derived medium, increased phospho-STAT3 in BBB cells (Fig. 6b, Supplementary Figure S13). When IL-6 was depleted from AC-conditioned medium, phospho-STAT3 decreased compared to the levels measured in BBB cells exposed to AC-conditioned medium. The opposite result was obtained in BBB monolayer exposed to IL-6-supplemented NS-conditioned medium compared to NS-conditioned medium (Fig. 6b, Supplementary Figure S13). To support the hypothesis that the IL-6/STAT3 axis mediated the transcriptional changes in ABC transporter and TJ proteins, we used STA-21, an inhibitor of STAT3 [35], which impairs STAT3 phosphorylation on Tyr705, dimerization and transcriptional activity [36]. STA-21 reduced STAT3 activation (Fig. 6c, Supplementary Figure S14a), increased Pgp, BCRP and TJ proteins (Fig. 6d, Supplementary Figure S14b) and mRNAs (Supplementary Figure S14c), reduced the permeability to DOX, MXR and DEXT in BBB monolayer treated with AC-conditioned medium and IL-6-supplemented NS-conditioned medium (Fig. 6e). Furthermore, STA-21 abrogated the TEER drop induced by AC-conditioned medium (Supplementary Figure S14d). STA-21 produced the same effects on BBB monolayer treated with DMEM enriched with IL-6 (Supplementary Figure S15a-d), confirming that the inhibition of IL-6/STAT3 axis made BBB more competent.

Notably, bypassing IL-6 and targeting directly STAT3 was sufficient to increase BBB competence: indeed, the PROTAC SD-36, specifically degrading STAT3 [37] (Supplementary Figure S16a-b), abrogated the effects of the AC-conditioned medium on BBB monolayer. SD-36 increased ABC transporter/TJ mRNAs (Supplementary Figure S16c), decreased the permeability to DOX, MXR and DEXT (Supplementary Figure S16d), and increased TEER compared to BBB cells exposed to AC-conditioned medium (Supplementary Figure S16e).

## Discussion

Although it is known that GBM cells release soluble factors that may impair BBB properties [30], the mechanisms underlying this event are not completely clear. Here, we demonstrated for the first time that the degree of differentiation or stemness of GBM cells influences BBB permeability.

We used co-cultures of human BBB cells and patient-derived GBM, cultured as stem cells/NS or differentiated cells/AC, to recapitulate in vitro the two extremes of the intratumor heterogeneity present at the GBM-BBB interface. In the presence of GBM cells, BBB cells showed a greater apical-to-basolateral permeability to dextran-70, which cannot cross BBB if TJ complexes are competent [38–40] (Fig. 1; Supplementary Figure S2). Accordingly, TJ proteins on BBB cells were downregulated in AC/BBB co-cultures compared to BBB cells alone. Unexpectedly, they were up-regulated in NS/BBB co-cultures despite in this condition the permeability to dextran-70 remained higher. This result indicates that TJ complexes are likely not competent in NS/BBB co-cultures, despite the gene up-regulation that may be interpreted as an unsuccessful compensatory event. Besides changes in TJs, we also observed an increase in the apical-to-basolateral permeability of doxorubicin and mitoxantrone, two typical substrates of Pgp and BCRP, in BBB monolayer co-cultured with GBM. Once again, AC increased permeability more than NS, and – similarly to what was observed with TJ proteins – these changes were mediated by a decreased transcription of ABC transporters. The increases in the permeability of doxorubicin and mitoxantrone were small but increases of this order of magnitude may have a translational significance since they have rescued the anti-GBM activity of doxorubicin in *vitro* and in vivo [41].

It has been often reported that CSCs of GBM are surrounded by a leaky and poorly competent BBB, creating a hypoxic and immunosuppressive environment that hinders effective drug delivery and efficacy [42]. This phenotype matches with our data showing that BBB monolayer becomes more permeant when co-cultured with NS compared to the BBB alone. However, if great attention has been paid to the CSC component of GBM since it is the most difficult to eradicate and the main cause of recurrence, to the best of our knowledge no works studied in-depth the impact that more differentiated GBM cells, which represent most tumor cells, have on BBB properties. The novelty of our work is the dissection of the effects of more differentiated cells and more stem-cell-like cells of GBM on BBB properties.

A previous work reported an increased permeability of brain microvascular endothelial cells co-cultured with GBM cells, caused by the down-regulation of occludin, ZO1, claudin-3 and claudin-5: the increase in permeability was smaller with low-grade astrocytoma (WHO grade II glioma) and anaplastic astrocytoma (WHO grade III glioma) compared to GBM (WHO IV glioma) [43]. The patient-derived samples used in the current work derive from grade IV gliomas: hence, it is expected that cells derived from these tumors induce a strong increase in BBB permeability. GBM is highly heterogeneous, showing the simultaneous presence of differentiated cells and CSCs in the same areas. Also, BBB properties are heterogeneous within GBM [44]. Indeed, the prevalence of one type of cells over the others may create small niches that differently impact on BBB properties, explaining some discrepancies between the results of the present study and past literature data. A recent single-cell analysis of BBB cells extracted from GBM revealed distinct transcriptomic profiles between clusters originating from the tumor bulk and clusters originating from the tumor’s peripheral area [44]. The clusters derived from the tumor bulk, which is rich in highly proliferative mesenchymal GBM CSCs [45], are characterized by elevated expression of markers related to neoangiogenesis and pro-inflammatory status. In contrast, the clusters derived from the tumor periphery, an area richer of proneural GBM CSCs [45], showed more differentiated phenotype and higher expression of TJ proteins and ABC transporters [44]. According to the phenotypic profile performed in the current study, we cannot discriminate if the NS have the genetic markers typical of mesenchymal-like or proneuronal-like GBM CSCs. Second, the AC and NS used have been generated ex vivo after their collection from tumor bulk: this is an obvious limitation when we compare in vivo and ex vivo co-culture models. In addition, while NS implanted orthotopically develop a GBM tumor, AC cells are not tumorigenic, as demonstrated by us [9] and others [46], making unfeasible the head-to-head comparison of the impact that AC and NS have on BBB in vivo. Far from representing real tumor biology, the present study is a proof-of-concept to shed light on possible crosstalk mechanisms between BBB and GBM cells that may influence BBB permeability.

Since BBB is a highly dynamic structure, whose properties are regulated by endothelial cells, pericytes and astrocytes [40] and their multiple crosstalks [41, 42]), we increased the complexity of our model to be closer to the physiological BBB.

Our simplest model, constituted only by GBM and endothelial cells, demonstrated that the BBB permeability relies on factors exchanged between these two components. To verify if this simplified model adequately recapitulates the biological complexity observed in vivo, we performed a head-to-head comparison between a monoculture-based model (endothelial cells only) and a triculture-based model (including endothelial cells, astrocytes and pericytes) (Fig. 3; Supplementary Figure S6). TEER and dextran-70 permeability were higher and lower, respectively, in the triculture-based model, indicating a higher TJ competence. By contrast, changes in doxorubicin and mitoxantrone permeability were negligible between the two models, suggesting that transport processes mediated by Pgp and BCRP are influenced primarily by interactions between tumor and endothelial cells rather than by the presence of pericytes and astrocytes.

Furthermore, it has been reported that culture medium alters BBB properties [47, 48]. For instance, hydrocortisone promotes BBB cell differentiation, increasing TJ protein expression (as occludin and claudin-5) and TEER [49, 50]. In the present work, hydrocortisone concentration was 0.5 µM when hCMEC/D3 cells were cultured alone and 0.20 µM when hCMEC/D3 cells were co-cultured with GBM cells and part of the BBB medium was replaced by GBM medium, which does not contain hydrocortisone. Both concentrations were above the concentration (0.18 µM) reported to reduce BBB permeability [50]. Therefore, we believe that the effects of hydrocortisone are maintained in BBB alone as well as in BBB co-cultured with GBM cells or medium. Other factors may be involved in modulating TJ expression, although in the case of HBMEC/ciβ cells growing in media with different sets of growth factors, only hydrocortisone had an impact on BBB properties [50]. Indeed, the lack of a perfect correspondence on TJ protein levels between GBM/BBB co-cultures and BBB exposed to the conditioned medium (Figs. 1 and 2; Supplementary Figures S2 and S5) may imply that contact factors, beyond soluble factors, also affect the expression of TJ proteins. As far as ABC transporters are concerned, they are modulated in the same way by AC-conditioned medium of AC GBM/BBB co-cultures. Conversely, with the NS-conditioned medium, which is richer in growth factors, we obtained higher interpatient variability: it is not unreasonable that each patient-derived tumor, characterized by different activity and expression of receptors and downstream pathways, had different sensitivity to specific growth factors. We recognize that this is another limitation of the present work. However, our experimental setting with rhIL-6 and NAb for IL-6 pointed out that the level of IL-6 is a key determinant to change BBB permeability, regardless of the culture medium surrounding hCMEC/D3 cells or specific growth factors.

Indeed, considering that neuroinflammation is a significant driver of GBM [51], we performed a high-throughput screening of the cytokines and chemokines present in GBM-conditioned medium, reasoning that they could be potentially good candidates mediating the changes in BBB permeability (Fig. 4; Supplementary Figure S7). TNF-α, MCP-1, IL-8 and IL-6 were higher in AC than in NS medium. All these cytokines have been implicated in the BBB disruption occurring in neurodegenerative disorders and ischemic stroke [52, 53]. It is known that IL-6 and IL-8 are produced by GBM cells [23, 54] and increase the permeability of BBB [30], but they have never been correlated with the degree of GBM differentiation. Since only IL-6 was confirmed to be significantly higher in AC than in NS medium in quantitative ELISA, we focused on this cytokine.

To clarify its role, we respectively added or depleted IL-6 in NS- or AC-conditioned medium co-cultured with BBB monolayer, to bring extracellular IL-6 at the same level in both settings (Fig. 5). IL-6-depleted AC-conditioned medium prevented the increase of BBB permeability, whereas IL-6-supplemented NS-conditioned medium produced the opposite effect. The release of IL-6 by U87 GBM cell line (around 200 pg/mL) was not sufficient to modulate BBB permeability in 4 h of co-culture, while the presence of activated microglia, simulating a neuroinflammatory situation, enhanced such release up to 400 pg/mL and increased BBB permeability by down-regulating TJ proteins via JAK2/STAT3 axis [55]. In our model, we did not include any microglial components, but only GBM cells with different degrees of differentiation. AC cells released IL-6 at the concentration of 200 pg/mL, but BBB cells were exposed to such concentration for 72 h. This condition was sufficient to activate the JAK/STAT3 axis and down-regulate TJ and ABC transporters in BBB cells, increasing the permeability in both the simplest BBB model (endothelial cells alone) and the triculture model (endothelial cells, astrocytes and pericytes). Comparing our data with those of Couto et al. [55], we can conclude that either a higher pulse of IL-6 for a short time (400 pg/mL for 4 h) or lower concentrations for a prolonged time (200 pg/mL for 72 h) produced the same molecular effects on BBB cells.

In microglia-GBM co-cultures, the use of an anti-IL-6 antibody re-establishes the normal barrier function [56]. Additionally, the systemic infusion of IL-6 NAb attenuates the increase in BBB permeability caused by ischemia [56], while high levels of IL-6 promote BBB disruption and decrease Pgp in Alzheimer’s disease [57].

Our results, obtained by using a neutralizing antibody against IL-6 or silencing IL-6 in AC, indicate that IL-6 is the main factor causing the differential changes in BBB permeability elicited by AC and NS. Mechanistically, IL-6 activated STAT3 in BBB cells (Fig. 5; Supplementary Figure S12), but only sporadic observations linked STAT3 with BBB dysfunction. For instance, during neuroinflammation, the activation of STAT3 in BBB cells downregulated β-catenin and ZO-1: BBB permeability was restored by depleting the medium of IL-6 or using the JAK inhibitor AG490, which blocked JAK/STAT3 axis [55]. Oncostatin M, a cytokine of IL-6 family, also induced BBB dysfunction by activating JAK/STAT3 signalling in bovine microvascular endothelial cells, while the JAK inhibitor ruxolitinib reversed its effect [58]. This pathway is evolutionarily conserved, since the inhibition of STAT3 signalling restores BBB dysfunctions also in Drosophila and mice [59].

In our GBM/BBB model, the IL-6/STAT3 axis simultaneously regulated ABC transporters and TJs in BBB cells, leading to a significant increase in the permeability to multiple substrates (Fig. 6; Supplementary Figure S15, Supplementary Figure S16). As further proof of the involvement of STAT3 in this process, we tested STA-21, a selective STAT3 inhibitor that has been successfully assessed in psoriatic patients (NCT01047943; https://clinicaltrials.gov/) [35]. STA-21 reversed IL-6 effects on BBB cells, by increasing ABC transporters and TJ proteins and reducing the permeability to their substrates. Similarly, targeting STAT3 with the first-in-class PROTAC SD-36 [37] counteracted the effects of the AC-conditioned medium.

Increasing IL-6 levels may improve the delivery of chemotherapeutic drugs within GBM mass, particularly in niches rich in CSCs where the BBB is less disrupted. IL-6 infusion is not easily feasible, because high serum levels of IL-6 are associated with side-effects known as cytokine release syndrome [60]. On the other hand, STAT3 is stimulated not only by IL-6 but also by several co-activators as non-coding RNAs [61]. Nanoparticles carrying non-coding RNAs directed to the basolateral side of BBB cells have been recently developed for GBM and neurodegenerative diseases [62, 63]. This approach could represent a step forward to achieve selective and localized activation of the IL-6/STAT3 axis on BBB cells, thereby improving chemotherapeutic drug delivery by down-regulating the most common drug efflux transporters.

## Conclusions

We demonstrated for the first time that the permeability of BBB is influenced by the degree of differentiation of GBM cells. The crosstalk between IL-6-producing GBM cells and BBB cells, which activate STAT3, simultaneously decreased ABC transporters and TJ proteins, thus increasing BBB permeability to different chemotherapeutic drugs virtually BBB-impermeant. The selective activation of the IL-6/STAT3 axis in specific areas of the GBM/BBB interface, particularly those rich in cancer stem cells and characterized by low disruption of BBB, may represent an innovative approach to enhance drug delivery, enlarge the spectrum of chemotherapeutic drugs used and achieve the pharmacological eradication of GBM.

## Electronic Supplementary Material

Below is the link to the electronic supplementary material.

Supplementary Material 1

## Data Availability

Data is provided within the manuscript or supplementary information files.

## Abbreviations

GBM: glioblastoma multiforme
CSCs: cancer stem cells
Pgp: P-glycoprotein
ABC: ATP Binding Cassette
BBB: blood-brain barrier
TJs: tight junctions
BCRP: breast cancer resistance protein
BAT: brain-adjacent-to-tumor
TME: tumor microenvironment
IL: interleukin
WHO: World Health Organization
hPE: human pericytes
hAS: human astrocytes
AC: adherent cells
NS: neurospheres
DOX: doxorubicin
MXR: mitoxantrone
DEXT: dextran 70-fluorescein isothiocyanate
NAb: neutralizing antibody
PROTAC: proteolysis targeting chimera
TEER: transendothelial electrical resistance
ZO-1: zonula occludens-1
STAT3: Signal Transducer and Activator of Transcription 3
CCL2/MCP1: chemokine (C-C motif) ligand 2/monocyte chemoattractant protein 1
CXCL8/IL-8: chemokine (C-X-C motif) ligand/interleukin-8
TNF-α: tumor necrosis factor-α

## References

[1] Alifieris C, Trafalis DT. <ArticleTitle Language=“En”>Glioblastoma multiforme: Pathogenesis and treatment. Pharmacol Ther. 2015;152:63–82. 25944528 10.1016/j.pharmthera.2015.05.005

[2] Grochans S, Cybulska AM, Simińska D, Korbecki J, Kojder K, Chlubek D, et al. Epidemiol Glioblastoma Multiforme–Literature Rev Cancers (Basel). 2022;14(10):2412. 10.3390/cancers14102412 PMC9139611 35626018

[3] Stupp R, Taillibert S, Kanner A, Read W, Steinberg DM, Lhermitte B, et al. Effect of Tumor-Treating Fields Plus Maintenance Temozolomide vs Maintenance Temozolomide Alone on Survival in Patients With Glioblastoma. JAMA. 2017;318:2306–16. 29260225 10.1001/jama.2017.18718 PMC5820703

[4] Zhao M, van Straten D, Broekman MLD, Préat V, Schiffelers RM. Nanocarrier-based drug combination therapy for glioblastoma. Theranostics. 2020;10(3):1355–72. 31938069 10.7150/thno.38147 PMC6956816

[5] Pinzon-Daza M, Campia I, Kopecka J, Garzon R, Ghigo D, Riganti C. Nanoparticle- and Liposome-carried Drugs: New Strategies for Active Targeting and Drug Delivery Across Blood-brain Barrier. Curr Drug Metab. 2013;14:625–40. 23869808 10.2174/1389200211314060001

[6] Tomar MS, Kumar A, Srivastava C, Shrivastava A. Elucidating the mechanisms of Temozolomide resistance in gliomas and the strategies to overcome the resistance. Biochim Biophys Acta Rev Cancer. 2021;1876(2):188616. 34419533 10.1016/j.bbcan.2021.188616

[7] DeCordova S, Shastri A, Tsolaki AG, Yasmin H, Klein L, Singh SK, et al. Molecular Heterogeneity and Immunosuppressive Microenvironment in Glioblastoma. Front Immunol. 2020;11:1402. 32765498 10.3389/fimmu.2020.01402 PMC7379131

[8] Gimple RC, Bhargava S, Dixit D, Rich JN. Glioblastoma stem cells: lessons from the tumor hierarchy in a lethal cancer. Genes Dev. 2019;33:591–609. 31160393 10.1101/gad.324301.119 PMC6546059

[9] Riganti C, Salaroglio IC, Caldera V, Campia I, Kopecka J, Mellai M, et al. Temozolomide downregulates P-glycoprotein expression in glioblastoma stem cells by interfering with the Wnt3a/glycogen synthase-3 kinase/β-catenin pathway. Neuro Oncol. 2013;15(11):1502–17. 23897632 10.1093/neuonc/not104 PMC3813413

[10] Salaroglio IC, Mujumdar P, Annovazzi L, Kopecka J, Mellai M, Schiffer D, et al. Carbonic anhydrase XII inhibitors overcome P-glycoprotein–mediated resistance to temozolomide in glioblastoma. Mol Cancer Ther. 2018;17:2598–609. 30254183 10.1158/1535-7163.MCT-18-0533

[11] Sundar SJ, Hsieh JK, Manjila S, Lathia JD, Sloan A. The role of cancer stem cells in glioblastoma. Neurosurg Focus. 2014;37(6):E6. 25434391 10.3171/2014.9.FOCUS14494

[12] Rathi S, Griffith JI, Zhang W, Zhang W, Oh JH, Talele S, et al. The influence of the blood–brain barrier in the treatment of brain tumors. J Intern Med. 2022;292:3–30. 35040235 10.1111/joim.13440

[13] Liebner S, Dijkhuizen RM, Reiss Y, Plate KH, Agalliu D, Constantin G. Functional morphology of the blood–brain barrier in health and disease. Acta Neuropathol. 2018;135(3):311–36. 29411111 10.1007/s00401-018-1815-1 PMC6781630

[14] Pinzón-Daza ML, Salaroglio IC, Kopecka J, Garzòn R, Couraud PO, Ghigo D, et al. The cross-talk between canonical and non-canonical Wnt-dependent pathways regulates P-glycoprotein expression in human blood-brain barrier cells. J Cerebr Blood Flow Metab. 2014;34:1258–69. 10.1038/jcbfm.2014.100 PMC4126086 24896565

[15] Elschot EP, Backes WH, Postma AA, Van Oostenbrugge RJ, Staals J, Rouhl RPW, et al. A Comprehensive View on MRI Techniques for Imaging Blood-Brain Barrier Integrity. Invest Radiol. 2021;56(1):10–9. 32932377 10.1097/RLI.0000000000000723

[16] Arvanitis CD, Ferraro GB, Jain RK. The blood–brain barrier and blood–tumor barrier in brain tumors and metastases. Nat Rev Cancer. 2020;20:26–41. 31601988 10.1038/s41568-019-0205-x PMC8246629

[17] Dréan A, Goldwirt L, Verreault M, Canney M, Schmitt C, Guehennec J, et al. Blood-brain barrier, cytotoxic chemotherapies and glioblastoma. Expert Rev Neurother. 2016;16:1285–300. 27310463 10.1080/14737175.2016.1202761

[18] Van Tellingen O, Yetkin-Arik B, de Gooijer MC, Wesseling P, Wurdinger T, de Vries HE. Overcoming the blood-brain tumor barrier for effective glioblastoma treatment. Drug Resist Updat. 2015;19:1–12. 25791797 10.1016/j.drup.2015.02.002

[19] Huang Z, Wong LW, Su Y, Huang X, Wang N, Chen H, et al. Blood-brain barrier integrity in the pathogenesis of Alzheimer’s disease. Front Neuroendocrinol. 2020;59:100857. 32781194 10.1016/j.yfrne.2020.100857

[20] Sweeney MD, Sagare AP, Zlokovic BV. Blood-brain barrier breakdown in Alzheimer disease and other neurodegenerative disorders. Nat Rev Neurol. 2018;14(3):133–50. 29377008 10.1038/nrneurol.2017.188 PMC5829048

[21] Abdullahi W, Tripathi D, Ronaldson PT. Blood-brain barrier dysfunction in ischemic stroke: targeting tight junctions and transporters for vascular protection. Am J Physiol Cell Physiol. 2018;315:343–56. 10.1152/ajpcell.00095.2018 PMC6171039 29949404

[22] Huang X, Hussain B, Chang J. Peripheral inflammation and blood–brain barrier disruption: effects and mechanisms. CNS Neurosci Ther. 2021;27:36–47. 33381913 10.1111/cns.13569 PMC7804893

[23] Albulescu R, Codrici E, Popescu ID, Mihai S, Necula LG, Petrescu D, et al. Cytokine patterns in brain tumor progression. Mediators Inflamm. 2013;2013:979748. 23864770 10.1155/2013/979748 PMC3707225

[24] Rochfort KD, Cummins PM. The blood–brain barrier endothelium: a target for pro-inflammatory cytokines. Biochem Soc Trans. 2015;43:702–6. 26551716 10.1042/BST20140319

[25] Weksler BB, Subileau EA, Perrière N, Charneau P, Holloway K, Leveque M, et al. Blood-brain barrier-specific properties of a human adult brain endothelial cell line. FASEB J. 2005;19(13):1872–4. 16141364 10.1096/fj.04-3458fje

[26] Barbar L, Jain T, Zimmer M, Kruglikov I, Sadick JS, Wang M, et al. CD49f Is a Novel Marker of Functional and Reactive Human iPSC-Derived Astrocytes. Neuron. 2020;107(3):436–e5312. 32485136 10.1016/j.neuron.2020.05.014 PMC8274549

[27] Grimaldi P, Lorenzati M, Ribodino M, Signorino E, Buffo A, Berchialla P. Predicting Astrocytic Nuclear Morphology with Machine Learning: A Tree Ensemble Classifier Study. Appl Sci. 2023;13:4289.

[28] Rizzi E, Deligne C, Dehouck L, Bilardo R, Sano Y, Shimizu F, Kanda T, Resmini M, Gosselet F, Dehouck MP, Mysiorek C. A Triple Culture Cell System Modeling the Human Blood-Brain Barrier. J Vis Exp. 2021;17710.3791/63134. 10.3791/63134 34927613

[29] Siflinger-Birnboim A, del Vecchio PJ, Cooper JA, Blumenstock FA, Shepard JM, Malik AB. Molecular Sieving Characteristics of the Cultured Endothelial Monolayer. J Cell Physiol. 1987;132(1):111–7. 3597548 10.1002/jcp.1041320115

[30] Mendes B, Marques C, Carvalho I, Costa P, Martins S, Ferreira D, et al. Influence of Glioma Cells on a New Co-Culture in Vitro Blood-Brain Barrier Model for Characterization and Validation of Permeability. Int J Pharm. 2015;490(1–2):94–101. 25981617 10.1016/j.ijpharm.2015.05.027

[31] Nilsson CL, Dillon R, Devakumar A, Shi SD, Greig M, Rogers JC, et al. Quantitative phosphoproteomic analysis of the STAT3/IL-6/HIF1α signaling network: an initial study in GSC11 glioblastoma stem cells. J Proteome Res. 2010;9(1):430–43. 19899826 10.1021/pr9007927

[32] Igelmann S, Neubauer HA, Ferbeyre G. STAT3 and STAT5 Activation in Solid Cancers. Cancers (Basel). 2019;11(10):1428. 31557897 10.3390/cancers11101428 PMC6826753

[33] Fasler-Kan E, Suenderhauf C, Barteneva N, Poller B, Gygax D, Huwyler J. Cytokine signaling in the human brain capillary endothelial cell line hCMEC/D3. Brain Res. 2010;1354:15–22. 20692239 10.1016/j.brainres.2010.07.077

[34] Takata F, Dohgu S, Matsumoto J, Machida T, Sakaguchi S, Kimura I, et al. Oncostatin M-induced blood-brain barrier impairment is due to prolonged activation of STAT3 signaling in vitro. J Cell Biochem. 2018;119(11):9055–63. 30076740 10.1002/jcb.27162

[35] Miyoshi K, Takaishi M, Nakajima K, Ikeda M, Kanda T, Tarutani M, et al. Stat3 as a therapeutic target for the treatment of psoriasis: a clinical feasibility study with STA-21, a Stat3 inhibitor. J Invest Dermatol. 2011;131(1):108–17. 20811392 10.1038/jid.2010.255

[36] Song H, Wang R, Wang S, Lin J. A low-molecular-weight compound discovered through virtual database screening inhibits Stat3 function in breast cancer cells. Proc Natl Acad Sci USA. 2005;102(13):4700–5. 15781862 10.1073/pnas.0409894102 PMC555708

[37] Bai L, Zhou H, Xu R, Zhao Y, Chinnaswamy K, McEachern D, et al. A Potent and Selective Small-Molecule Degrader of STAT3 Achieves Complete Tumor Regression In Vivo. Cancer Cell. 2019;36(5):498–511. 31715132 10.1016/j.ccell.2019.10.002 PMC6880868

[38] Watkins S, Robel S, Kimbrough IF, Robert SM, Ellis-Davies G, Sontheimer H. Disruption of astrocyte-vascular coupling and the blood-brain barrier by invading glioma cells. Nat Commun. 2014;5:4196. 24943270 10.1038/ncomms5196 PMC4127490

[39] Lin M, Zhu L, Wang J, Xue Y, Shang X. miR-424-5p maybe regulate blood-brain barrier permeability in a model in vitro with Abeta incubated endothelial cells. Biochem Biophys Res Commun. 2019;517(3):525–31. 31375213 10.1016/j.bbrc.2019.07.075

[40] Zhao Y, Gan L, Ren L, Lin Y, Ma C, Lin X. Factors influencing the blood-brain barrier permeability. Brain Res. 2022;1788:147937. 35568085 10.1016/j.brainres.2022.147937

[41] Salaroglio IC, Abate C, Rolando B, Battaglia L, Gazzano E, Colombino E, et al. Validation of Thiosemicarbazone Compounds as P-Glycoprotein Inhibitors in Human Primary Brain-Blood Barrier and Glioblastoma Stem Cells. Mol Pharm. 2019;16(8):3361–73. 31265310 10.1021/acs.molpharmaceut.9b00018

[42] Auffinger B, Spencer D, Pytel P, Ahmed AU, Lesniak MS. The role of glioma stem cells in chemotherapy resistance and glioblastoma multiforme recurrence. Expert Rev Neurother. 2015;15(7):741–52. 26027432 10.1586/14737175.2015.1051968 PMC4830899

[43] Ishihara H, Kubota H, Lindberg RLP, Leppert D, Gloor SM, Errede M, et al. Endothelial Cell Barrier Impairment Induced by Glioblastomas and Transforming Growth Factor A 2 Involves Matrix Metalloproteinases and Tight Junction Proteins. J Neuropathol Exp Neurol. 2008;67(5):435–48. 18431253 10.1097/NEN.0b013e31816fd622

[44] Xie Y, He L, Lugano R, Zhang Y, Cao H, He Q, et al. Key molecular alterations in endothelial cells in human glioblastoma uncovered through single-cell RNA sequencing. JCI Insight. 2021;6(15):e150861. 34228647 10.1172/jci.insight.150861 PMC8410070

[45] Wang L, Babikir H, Müller S, Yagnik G, Shamardani K, Catalan F, et al. The Phenotypes of Proliferating Glioblastoma Cells Reside on a Single Axis of Variation. Cancer Discov. 2019;9(12):1708–19. 31554641 10.1158/2159-8290.CD-19-0329 PMC7161589

[46] Lathia JD, Gallagher J, Myers JT, Li M, Vasanji A, McLendon RE, et al. Direct in vivo evidence for tumor propagation by glioblastoma cancer stem cells. PLoS ONE. 2011;6(9):e24807. 21961046 10.1371/journal.pone.0024807 PMC3178553

[47] Schneider SW, Ludwig T, Tatenhorst L, Braune S, Oberleithner H, Senner V, et al. Glioblastoma cells release factors that disrupt blood–brain barrier features. Acta Neuropathol. 2004;107(3):272–6. 14730455 10.1007/s00401-003-0810-2

[48] Neuhaus W, Wirth M, Plattner VE, Germann B, Gabor F, Noe CR. Expression of Claudin-1, Claudin-3 and Claudin-5 in human blood-brain barrier mimicking cell line ECV304 is inducible by glioma-conditioned media. Neurosci Lett. 2008;446(2–3):59–64. 18817843 10.1016/j.neulet.2008.09.025

[49] Förster C, Burek M, Romero IA, Weksler B, Couraud PO, Drenckhahn D. Differential effects of hydrocortisone and TNFalpha on tight junction proteins in an in vitro model of the human blood-brain barrier. J Physiol. 2008;586(7):1937–49. 18258663 10.1113/jphysiol.2007.146852 PMC2375735

[50] Furihata T, Kawamatsu S, Ito R, Saito K, Suzuki S, Kishida S, et al. Hydrocortisone enhances the barrier properties of HBMEC/ciβ, a brain microvascular endothelial cell line, through mesenchymal-to-endothelial transition-like effects. Fluids Barriers CNS. 2015;12:7. 25763180 10.1186/s12987-015-0003-0 PMC4355132

[51] Sim J, Park J, Moon JS, Lim J. Dysregulation of inflammasome activation in glioma. Cell Commun Signal. 2023;21(1):239. 37723542 10.1186/s12964-023-01255-5 PMC10506313

[52] Nishioku T, Matsumoto J, Dohgu S, Sumi N, Miyao K, Takata F, et al. Tumor Necrosis Factor-α Mediates the Blood–Brain Barrier Dysfunction Induced by Activated Microglia in Mouse Brain Microvascular Endothelial Cells. J Pharmacol Sci. 2010;112(2):251–4. 20118615 10.1254/jphs.09292sc

[53] Dimitrijevic OB, Stamatovic SM, Keep RF. AndjelkovicAV. Effects of the Chemokine CCL2 on Blood-Brain Barrier Permeability during Ischemia-Reperfusion Injury. J Cerebr Blood Flow Metab. 2006;26(6):797–810. 10.1038/sj.jcbfm.9600229 16192992

[54] Okawa S, Gagrica S, Blin C, Ender C, Pollard SM, Krijgsveld J. Proteome and Secretome Characterization of Glioblastoma-Derived Neural Stem Cells. Stem Cells. 2017;35(4):967–80. 27870168 10.1002/stem.2542 PMC6135235

[55] Couto M, Coelho-Santos V, Santos L, Fontes-Ribeiro C, Silva AP, Gomes CMF. The interplay between glioblastoma and microglia cells leads to endothelial cell monolayer dysfunction via the interleukin-6-induced JAK2/STAT3 pathway. J Cell Physiol. 2019;234(11):19750–60. 30937892 10.1002/jcp.28575

[56] Zhang J, Sadowska GB, Chen X, Park SY, Kim JE, Bodge CA, et al. Anti-IL-6 neutralizing antibody modulates blood-brain barrier function in the ovine fetus. FASEB J. 2015;29(5):1739–53. 25609424 10.1096/fj.14-258822 PMC4771067

[57] Rothaug M, Becker-Pauly C, Rose-John S. The role of interleukin-6 signaling in nervous tissue. Biochim Biophys Acta. 2016;1863(6PtA):1218–27. 27016501 10.1016/j.bbamcr.2016.03.018

[58] Takata F, Dohgu S, Sakaguchi S, Sakai K, Yamanaka G, Iwao T, et al. Oncostatin-M-Reactive Pericytes Aggravate Blood-Brain Barrier Dysfunction by Activating JAK/STAT3 Signaling In Vitro. Neuroscience. 2019;422:12–20. 31705893 10.1016/j.neuroscience.2019.10.014

[59] Kim J, Chuang HC, Wolf NK, Nicolai CJ, Raulet DH, Saijo K, et al. Tumor-induced disruption of the blood-brain barrier promotes host death. Dev Cell. 2021;56(19):2712–21. 34496290 10.1016/j.devcel.2021.08.010 PMC8511098

[60] Lee EY, Jakubovic BD. Interleukin-6 and cytokine release syndrome: A new understanding in drug hypersensitivity reactions. Ann Allergy Asthma Immunol. 2023;130(2):178–84. 36343890 10.1016/j.anai.2022.10.025

[61] Yang L, Lin S, Xu L, Lin J, Zhao C, Huang X. Novel activators and small-molecule inhibitors of STAT3 in cancer. Cytokine Growth Factor Rev. 2019;49:10–22. 31677966 10.1016/j.cytogfr.2019.10.005

[62] Singh RR, Mondal I, Janjua T, Popat A, Kulshreshtha R. Engineered smart materials for RNA based molecular therapy to treat Glioblastoma. Bioact Mater. 2023;33:396–423. 38059120 10.1016/j.bioactmat.2023.11.007 PMC10696434

[63] Israel LL, Sun T, Braubach O, Cox A, Shatalova ES, Rashid HM, et al. β-Amyloid targeting nanodrug for neuron-specific delivery of nucleic acids in Alzheimer’s disease mouse models. J Control Release. 2023;361:636–58. 37544515 10.1016/j.jconrel.2023.08.001

