# Supplementary material for: Blood-brain barrier permeability increases with the differentiation of glioblastoma cells in vitro
Source: Fluids Barriers CNS. 2024 Nov 1;21:89. doi: 10.1186/s12987-024-00590-0 (PMC11529439; doi:10.1186/s12987-024-00590-0)
Supplement: Supplementary file 1 — Supplementary Material 1 [file 12987_2024_590_MOESM1_ESM.pdf]

## Supplemental material

## Supplemental Tables

# Table S1. Patient clinical, pathologic and genetic data

| | **Patient #1** | **Patient #2** | **Patient #3** |
|---|---|---|---|
| Age at diagnosis | 57 | 53 | 61 |
| Sex | M | F | M |
| Histologic grade | IV | IV | IV |
| Therapy | Surgery + radiotherapy + chemotherapy | Surgery + radiotherapy + chemotherapy | Surgery + radiotherapy + chemotherapy |
| Time to recurrence (months) | 7 | 6 | 9 |
| Post-recurrence therapy | Reresection + radiotherapy | Radiotherapy | Radiotherapy + chemotherapy |
| Overall survival (months) | 12 | 9 | 18 |
| MGMT status | Partially methylated | Fully unmethylated | Fully unmethylated |
| EGFR status | Not amplified | Not amplified | Amplified |
| IDH1 status | Mutated (395G>A) | Mutated (395G>A) | Wild-type |
| IDH2 status | Wild-type | Wild-type | Wild-type |
| TP53 | Wild-type | Mutated (380C>T) | Wild-type |
| 1p/19q codeletion | Present | Absent | Absent |

Demographic, pathologic, clinical and genetic data of the patients analyzed in the study. Time to recurrence: time between the surgery and the tumor relapse at magnetic resonance imaging. Overall survival: time between diagnosis and patient death (from: Salaroglio IC et al. Mol Cancer Ther. 2018; 17: 2598-609).

**Table S2. Phenotypic characterization of patient-derived differentiated cells and stem cells**

| Markers | #1<br>AC | #1<br>NS | #2<br>AC | #2<br>NS | #3<br>AC | #3<br>NS |
|---|---|---|---|---|---|---|
| CD133 | - | - | - | + | - | -/+ |
| Nestin | - | + | - | ++ | - | + |
| Musashi-1 | - | + | - | ++ | - | + |
| GFAP | + | - | + | - | + | - |
| Gal-C | + | - | +/- | - | + | - |
| Nanog | 1.3% | 98.7% | 2.7% | 97.3% | 1.8% | 98.2% |
| Oct4 | 1.2% | 98.8% | 1.4% | 98.6% | 1.6% | 98.4% |
| SOX2 | 2.4% | 97.6% | 2.2% | 97.8% | 1.7% | 98.3% |
| ABCG2 | 3.4% | 96.6% | 5.1% | 94.9% | 1.9% | 98.1% |
| ALDH $^{bright}$ | 0.6% | 99.4% | 0.1% | 99.9% | 1.4% | 98.6% |

Differentiated/adherent cells (AC) and stem cell/neurospheres (NS) from #1, #2, #3 patients were analyzed by immunofluorescence microscopy for the neural stemness markers CD133, nestin, Musashi-1, and for the neural differentiation markers glial fibrillary acidic protein (GFAP) and galactocerebroside (Gal-C). Positivity of staining were graded as it follows: -: undetectable; +/-: low expression (< 25% cells positive); +: moderate expression (26%-74% cells positive); ++: high expression (> 75% cells positive). The percentage of Nanog-, Oct4-, SOX2-, ABCG2- and aldehyde dehydrogenase (ALDH)$^{bright}$ -positive cells, additional markers of stemness, was determined by flow cytometry and expressed as percentage of positive cells in the whole population (from: Salaroglio IC et al. Mol Cancer Ther. 2018; 17: 2598-609)

**Supplementary Figures**

# Figure S1

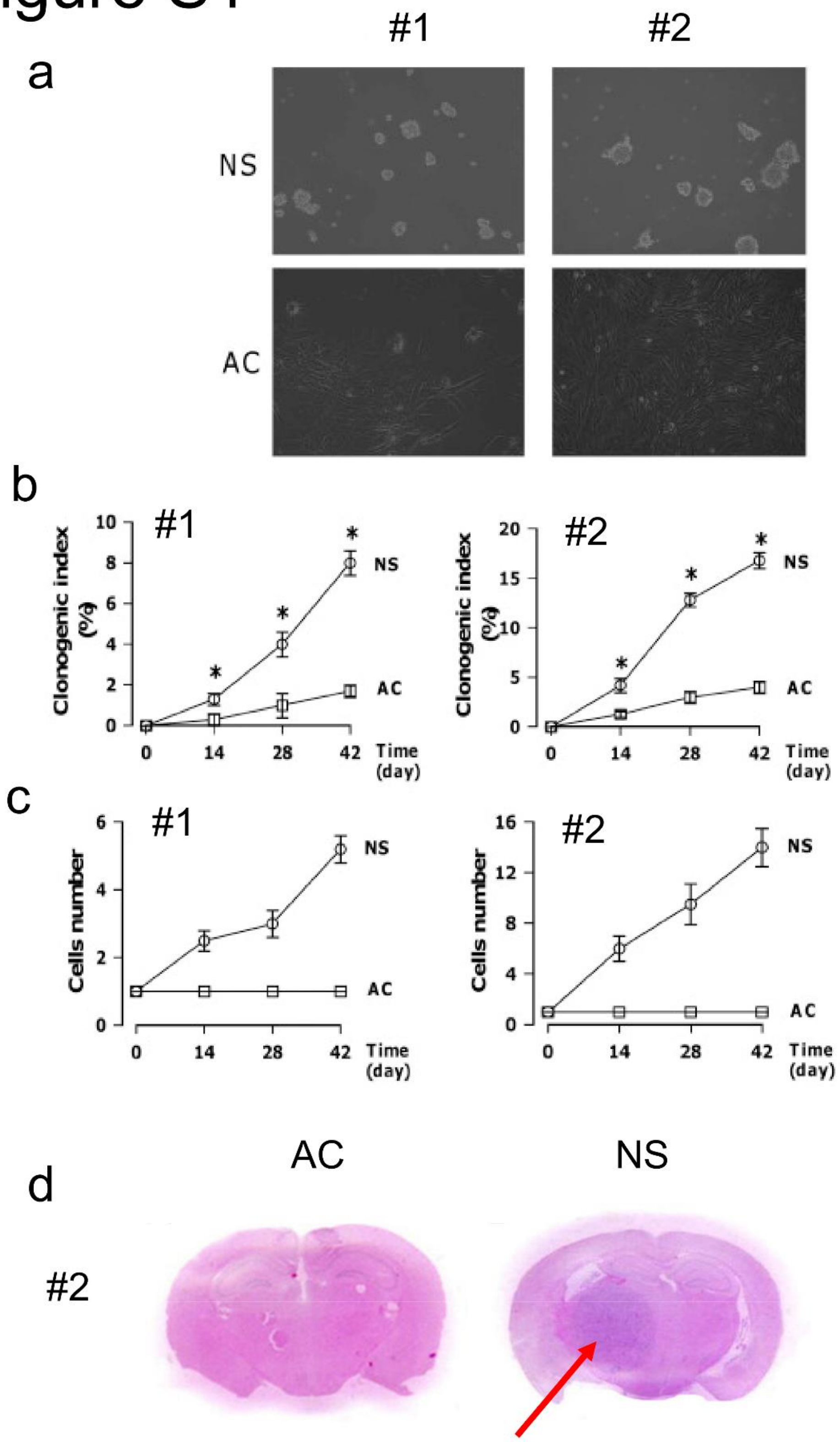

**Figure S1. Characterization of GBM neurospheres (NS) cells and adherent cells (AC). a.** Morphologic analysis of #1, #2 GBM cells, cultured as NS or Acs, and analysed by bright field microscope. Magnification: 100 × objective (0.52 numerical aperture); 10 × ocular lens. Bar: 20 μM. **b.** Clonogenic assay. NS or AC were seeded at a density of 100 cells/well; the spheres or adherent colonies were counted on days 14, 28, and 42. Data are presented as means ± SD (n= 6). Significance of NS vs AC: *p< 0.01. **c.** Self-renewal assay. NS or AC were diluted and seeded at a density of 1 cell/well; cells were counted on days 14, 28, and 42. Data are presented as means ± SD (n = 10). Since AC had a value of 1 cell ± 0 at each time point, statistical analysis was not performed. **d.** *In vivo* tumorigenicity**.** Hematoxilin-eosin staining of formalin-fixed paraffin embedded (FFPE) mouse brain coronal section after intracranial implant of #2 NS or AC. The images are representative of 1 mouse out of 4 per each group. Red arrow: tumor mass. (from: Riganti C et al., Neuro Oncol. 2013;15(11):1502-17).

# Figure S2

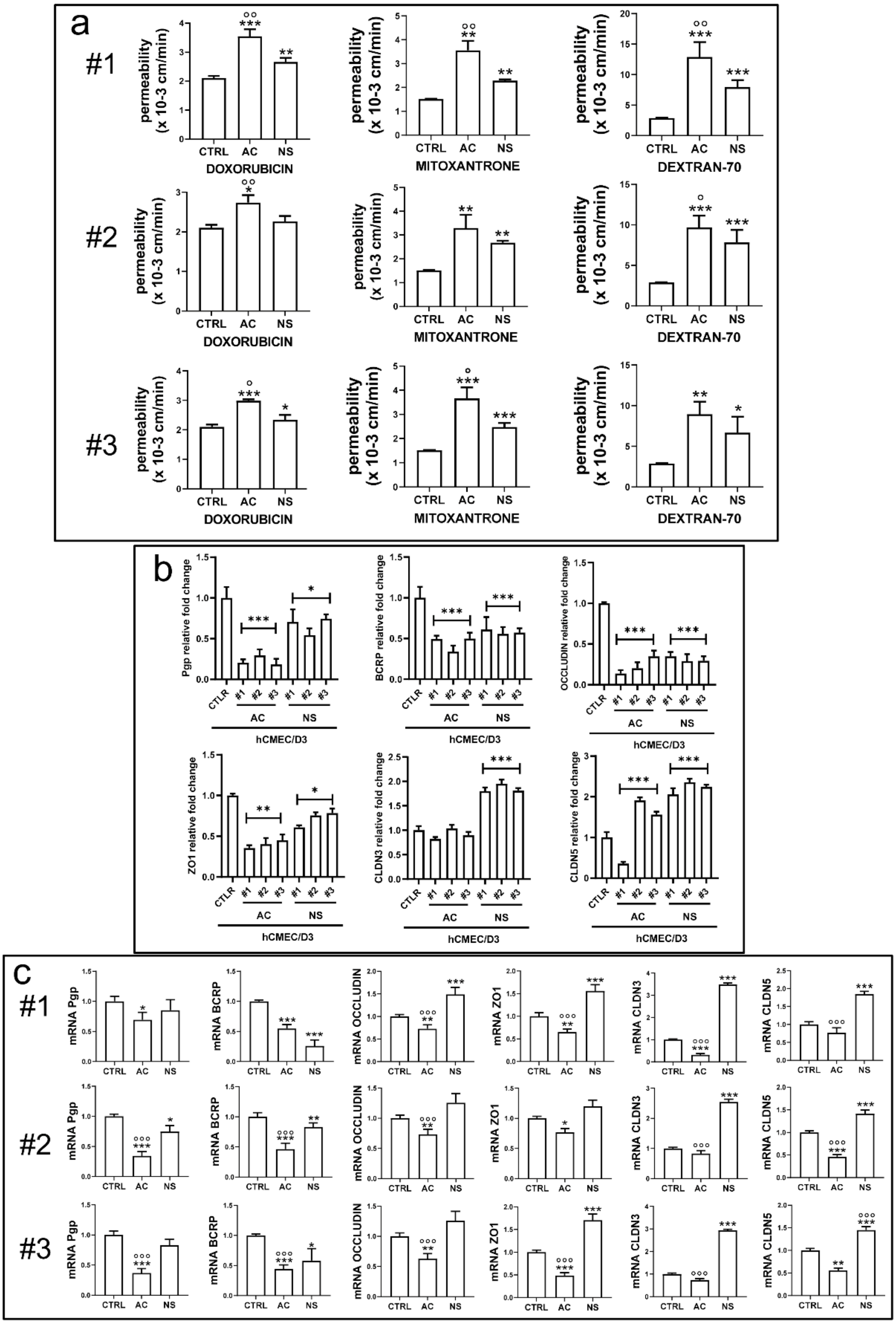

a
#1
#2
#3
permeability (x 10-3 cm/min)
CTRL
AC
NS
DOXORUBICIN
MITOXANTRONE
DEXTRAN-70
b
Pgp relative fold change
BCRP relative fold change
OCCLUDIN relative fold change
ZO1 relative fold change
CLDN3 relative fold change
CLDN5 relative fold change
CTLR
#1
#2
#3
AC
NS
hCMEC/D3
c
#1
#2
#3
mRNA Pgp
mRNA BCRP
mRNA OCCLUDIN
mRNA ZO1
mRNA CLDN3
mRNA CLDN5
CTRL
AC
NS

**Figure S2. Disaggregated data of permeability assays, ABC transporter expression and tight junction-related proteins of Figure 1**

Human blood-brain barrier (BBB) hCMEC/D3 cells were grown up to confluence in Transwell inserts. After 7 days, glioblastoma (GBM) cells derived from #1, #2, #3 patient, as differentiated/adherent cells (AC) or stem cell/neurospheres (NS), were added in the lower chamber for 72 h, then the medium in the upper and lower chamber was replaced and co-cultures were used for the experimental assays. A Transwell containing BBB cells only, grown for 7 days, was used as control (CTRL). **a.** Permeability assays in co-cultures with GBM cells of patients #1, #2 and #3. Data are presented as means ± SD (n=3 independent experiments; each experimental point was performed in technical duplicates). *p<0.05, **p<0.01, ***p< 0.001: AC/NS vs CTRL; °p<0.05, °°p<0.01: AC vs NS. **b.** Densitometric analysis of immunoblot of Figure 1**c**. The ratio "band density of protein of interest/housekeeping protein" in CTRL cells was considered 1. Results were the ratio between "band density of protein of interest/housekeeping protein" in each experimental condition, expressed as fraction of the CTRL ratio. *p<0.05, **p<0.01, ***p< 0.001: AC/NS vs CTRL. **c**. qRT-PCR of ABC transporter and tight junction (TJ) genes in BBB cells, in co-cultures with GBM cells of patients #1, #2 and #3. Data are presented as means ± SD (n=3 independent experiments; each experimental point was performed in technical triplicates). *p<0.05, **p<0.01, ***p< 0.001: AC/NS vs CTRL; °°°p<0.001: AC vs NS.

# Figure S3

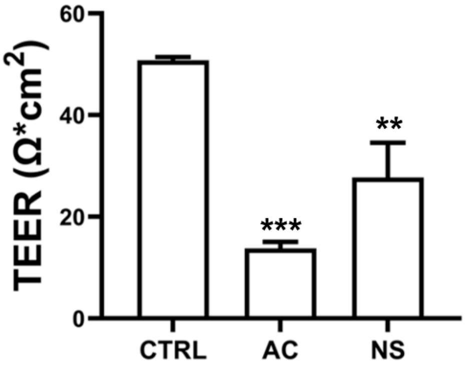


**Figure S3. TEER values of blood-brain barrier in the presence of differentiated and stem cells.** Human blood-brain barrier (BBB) hCMEC/D3 cells were grown up to confluence in Transwell inserts. After 7 days, glioblastoma cells derived from patients #1, #2, #3, as differentiated/adherent cells (AC) or stem cell/neurospheres (NS), were added in the lower chamber for 72 h. A Transwell containing BBB cells only, grown for 7 days, was used as control (CTRL). After the co-culture, TEER value was measured. The results are mean ± SD (n=3 independent experiments; each experimental point was performed in technical duplicates). **p< 0.01 ***p< 0.001: AC/NS vs CTRL.

# Figure S4

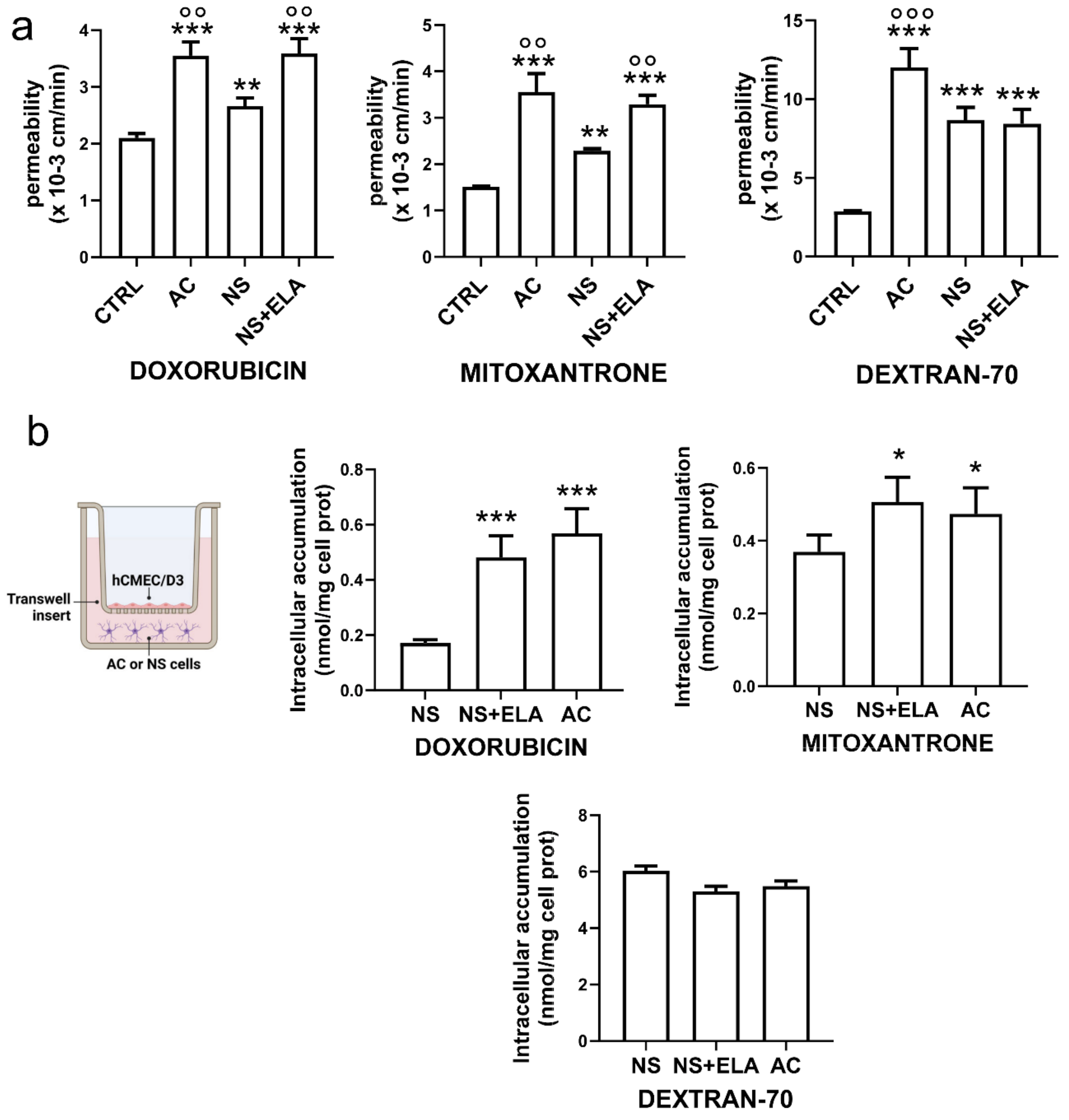


**Figure S4. The increased permeability of doxorubicin and mitoxantrone induced by AC/BBB co-cultures was mimicked by elacridar in NS/BBB co-cultures.** hCMEC/D3 cells were grown for 7 days up to confluence in Transwell insert (CTRL), then incubated with AC and NS from patient #2. After 72 h co-culture, 5 µM doxorubicin, 10 µM mitoxantrone or 2 µM dextran 70-fluorescein isothiocyanate were added for 3 h. When indicated, 2 µM of the elacridar (ELA), a dual Pgp/BCRP inhibitor, was co-incubated in the lower chamber where NS were present. **a.** Permeability assays. The compounds recovered from the medium of the lower chamber were measured fluorometrically. Data are presented as means ± SD (n=3 independent experiments; each experimental point was performed in technical duplicates). **p<0.01, ***p< 0.001: AC/NS vs CTRL; °°p<0.01,

°°°p<0.001: AC vs NS. **b**. Fluorometric quantification of intracellular doxorubicin, mitoxantrone and dextran 70-fluorescein isothiocyanate in AC and NS. Data are presented as means ± SD (n=3 independent experiments; each experimental point was performed in technical duplicates). *p<0.05, ***p< 0.001: AC/NS+ELA vs NS.

# Figure S5

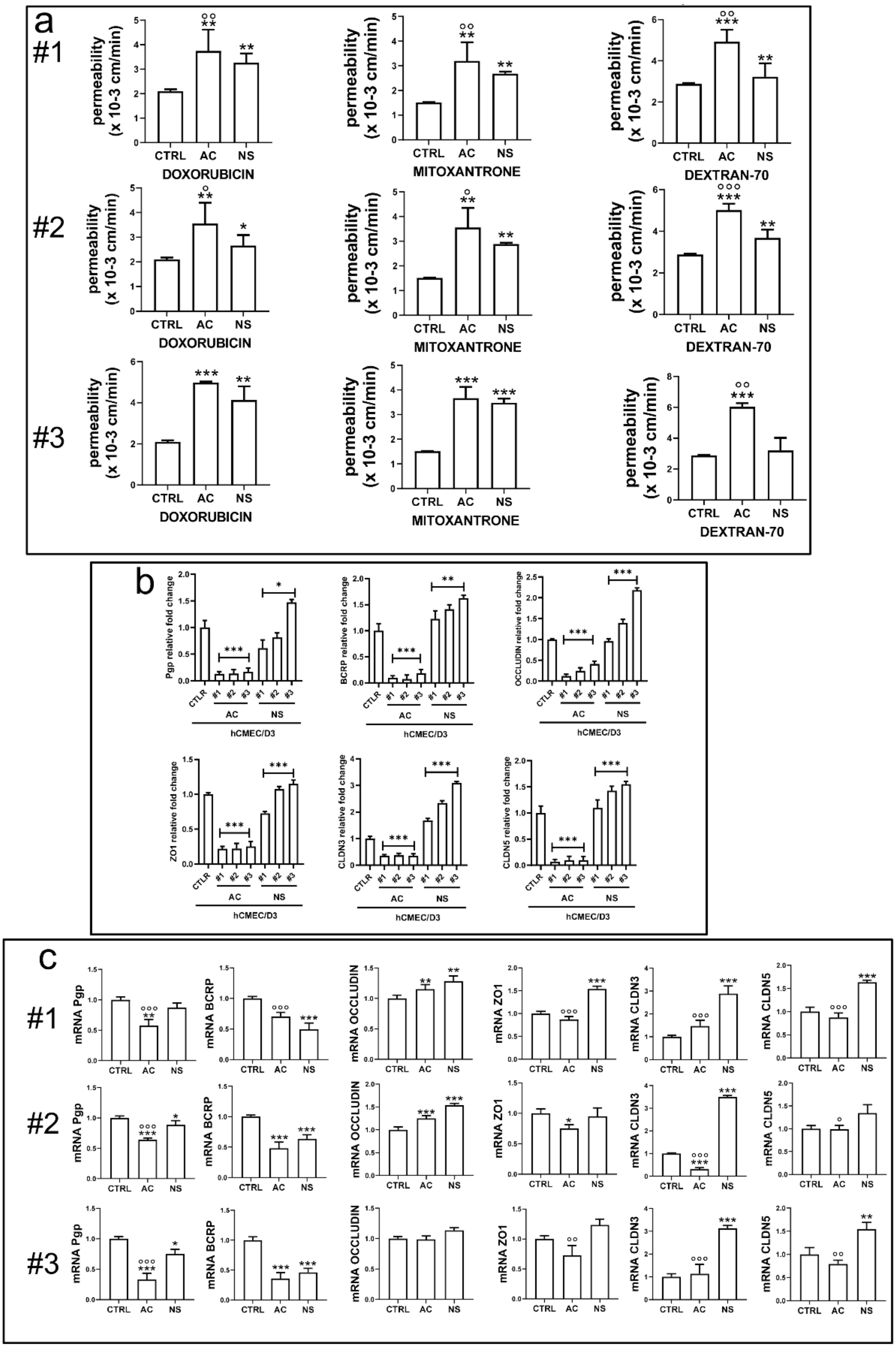
a
#1
#2
#3
permeability (x 10-3 cm/min)
CTRL AC NS
DOXORUBICIN
MITOXANTRONE
DEXTRAN-70
b
Pgp relative fold change
BCRP relative fold change
OCCLUDIN relative fold change
ZO1 relative fold change
CLDN3 relative fold change
CLDN5 relative fold change
CTLR #1 #2 #3 #1 #2 #3
AC NS
hCMEC/D3
c
mRNA Pgp
mRNA BCRP
mRNA OCCLUDIN
mRNA ZO1
mRNA CLDN3
mRNA CLDN5

**Figure S5. Disaggregated data of permeability assays, ABC transporter expression and tight junction proteins of Figure 2.** Human blood-brain barrier (BBB) hCMEC/D3 cells were grown up to confluence in Transwell inserts. After 7 days, the conditioned medium derived from a 5-day culture of glioblastoma (GBM) cells of patients #1, #2 and #3, as medium of differentiated/adherent cells (AC) or stem cell/neurospheres (NS), was added in the lower chamber for 72 h, then the medium in the upper and lower chamber was replaced and the cultures were used for the experimental assays. A Transwell containing BBB cells only, grown for 7 days, was used as control (CTRL). **a.** Permeability assays in cultures with the medium derived from GBM cells of patients #1, #2 and #3. Data are presented as means ± SD (n=3 independent experiments; each experimental point was performed in technical duplicates). *p<0.05, **p<0.01, ***p< 0.001: AC/NS vs CTRL; °p<0.05, °°p<0.01, °°°p<0.001: AC vs NS. **b.** Densitometric analysis of immunoblot of Figure 2**c**. The ratio “band density of protein of interest/housekeeping protein” in CTRL cells was considered 1. Results were the ratio between “band density of protein of interest/housekeeping protein” in each experimental condition, expressed as fraction of the CTRL ratio. *p<0.05, **p<0.01, ***p< 0.001: AC/NS vs CTRL. **c**. qRT-PCR of ABC transporter and tight junction (TJ) genes in BBB cells, in cultures with the medium derived from GBM cells of patients #1, #2 and #3. Data are presented as means ± SD (n=3 independent experiments; each experimental point was performed in technical triplicates). *p<0.05, **p<0.01, ***p<0.001: AC/NS vs CTRL; °p<0.05, °°p<0.01, °°°p<0.001: AC vs NS.

Figure S6

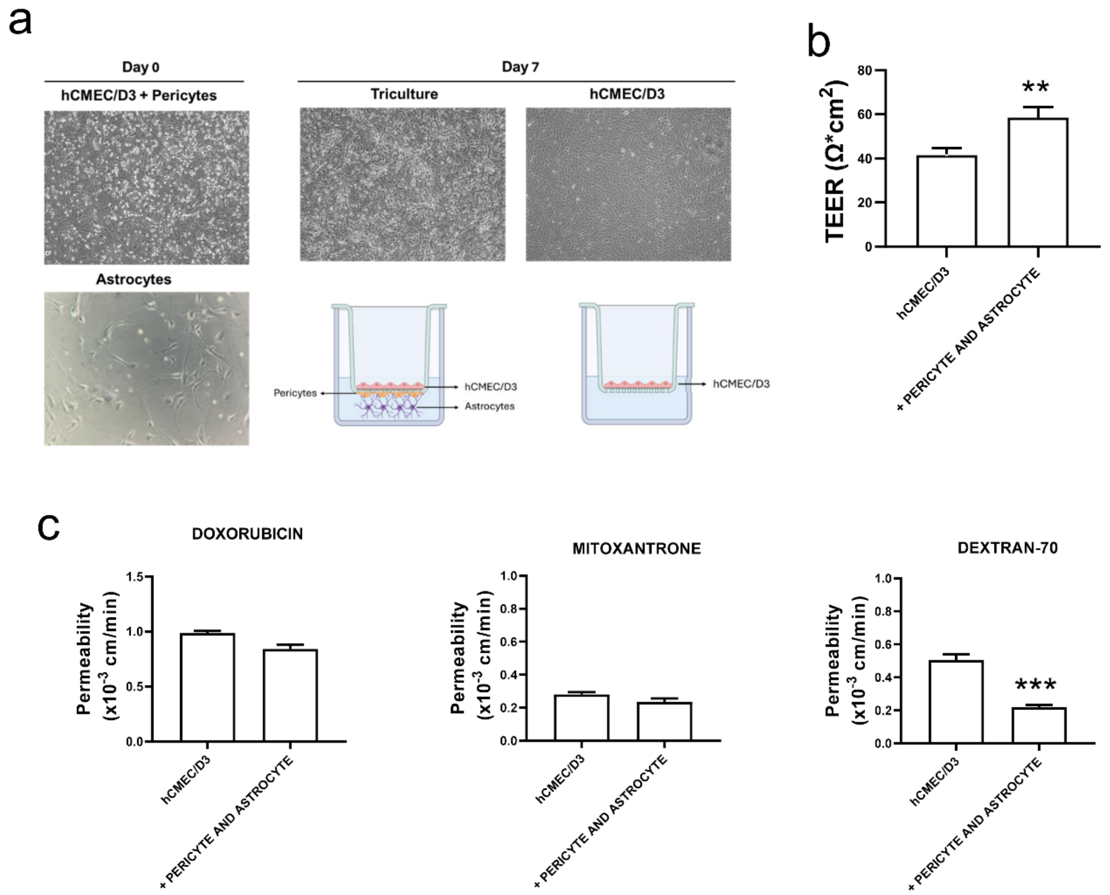


**Figure S6. Comparison between triculture-based and monoculture-based BBB model**

Triculture-based BBB was performed by seeding hCMEC/D3 cells in the Transwell insert, human pericytes in the reverted side of the insert and human astrocytes in the lower chamber. **a.** Bright field microscope images of hCMEC/D3+pericytes (insert) and astrocytes (lower chamber) at day 0 (when the triculture was assembled), of triculture-based BBB model and hCMEC/D3-based BBB model at day 7 (when BBB was competent). **b**. TEER values in hCMEC/D3 alone or hCMEC/D3 plus pericytes and astrocytes. Data are presented as mean ± SD (n=3 independent experiments; each experimental point was performed in technical duplicates). $^{**}p < 0.01$: hCMEC/D3 alone vs hCMEC/D3 plus pericytes and astrocytes. **c**. Permeability assays. 5 µM doxorubicin, 10 µM mitoxantrone or 2 µM dextran 70-fluorescein isothiocyanate were added in the Transwell insert. After 3 h, the compounds recovered from the medium of the lower chamber were measured fluorometrically. Data are presented as means ± SD (n=3 independent experiments; each experimental point was performed in technical duplicates). $^{***}p < 0.001$: hCMEC/D3 alone vs hCMEC/D3 plus pericytes and astrocytes.

# Figure S7

| | A | B | C | D | E | F | G | H | I | J | K |
|---|---|---|---|---|---|---|---|---|---|---|---|
| **1** | Pos | Pos | Pos | Pos | Neg | Neg | ENA-78 | GCSF | GM-CSF | GRO | GRO-α |
| **2** | I-309 | IL-1 α | IL-1β | IL-2 | IL-3 | IL-4 | IL-5 | IL-6 | IL-7 | IL-8 | IL-10 |
| **3** | IL-12 P40/p70 | IL-13 | IL-15 | IFN-γ | MCP-1 | MCP-2 | MCP-3 | MCSF | MDC | MIG | MIP-1b |
| **4** | MIP-1δ | RANTES | SCF | SDF-1 | TARC | TGF- β1 | TNF-α | TNF-β | EGF | IGF-1 | Angiogenin |
| **5** | Oncostatin M | Thrombopoietin | VEGF | PDGF-BB | Leptin | BDNF | BLC | Ck β 8-1 | Eotaxin | Eotaxin-2 | Eotaxin-3 |
| **6** | FGF-4 | FGF-6 | FGF-7 | FGF-9 | Flt-3 Ligand | Fractalkaline | GCP-2 | GDNF | HGF | IGFBP-1 | IGFBP-2 |
| **7** | IGFBP-3 | IGFBP-4 | IL-16 | IP-10 | LIF | LIGHT | MCP-4 | MIF | MIP-3 | NAP-2 | NT-3 |
| **8** | NT-4 | Osteopontin | Ostoprotegerin | PARC | PIGF | TGF-β2 | TGF-β3 | TIMP-1 | TIMP-2 | Pos | Pos |

**Figure S7. Schematic representation of cytokine position in the cytokine array**. Squares identify the differentially expressed cytokines in all patients. Red: IL-6; yellow: IL-8, TNF-α, MCP-1.

Figure S8

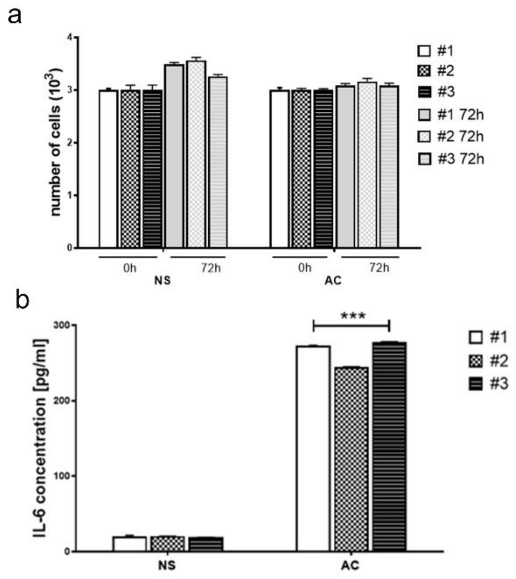


**Figure S8. Comparison of proliferation and IL-6 released by AC and NS GBM cells growing in the same culture conditions.** 3000 AC and NS cells from patients #1, #2 and #3 were seeded in 24 well plates coated by rat collagen thin layer for 72 h. **a.** The number of viable cells was evaluated with the Trypan blue exclusion staining and microscope analysis. Data are presented as mean ± SD (n=3 independent experiments; each experimental point was performed in technical duplicates). **b.** ELISA detection of IL-6 in AC- or NS-derived medium. Data are presented as mean ± SD (n=3 independent experiments; each experimental point was performed in technical duplicates). ***$p < 0.001$: AC vs NS

# Figure S9

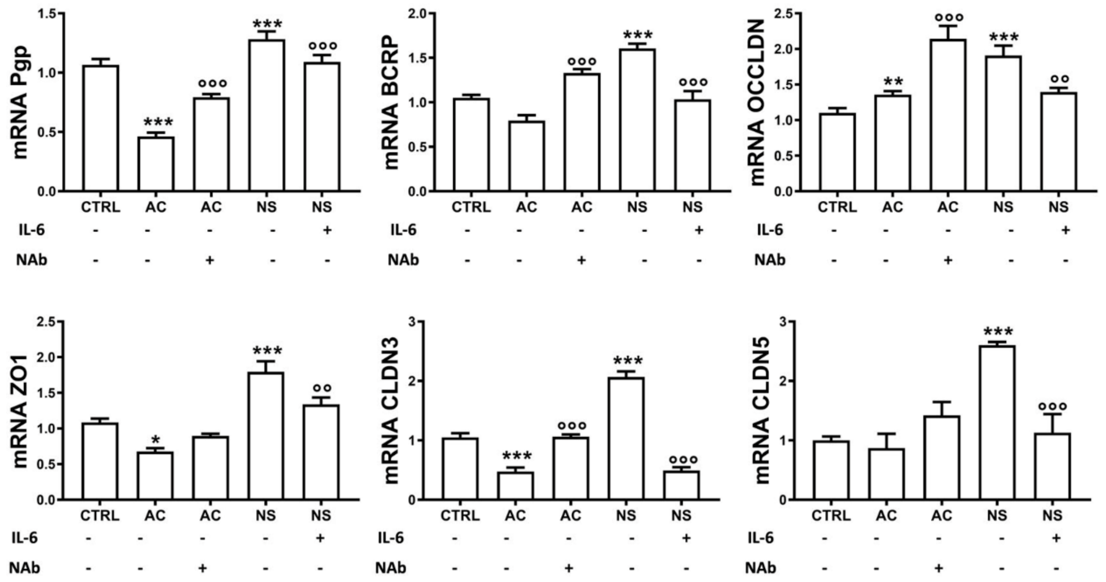


**Figure S9. Changes in ABC transporter and tight junction mRNAs in response to IL-6.** Human blood-brain barrier (BBB) hCMEC/D3 cells were grown up to confluence in Transwell inserts. After 7 days, the conditioned medium derived from a 5-day culture of glioblastoma (GBM) cells of patient #2, as medium of differentiated/adherent cells (AC) or stem cell/neurospheres (NS), was added in the lower chamber for 72 h. Recombinant human IL-6 (200 pg/mL) was added in NS-conditioned medium, a neutralizing IL-6 antibody (Nab, 1/400) was added in AC-conditioned medium. Then the medium in the upper and lower chamber was replaced and the cultures were used for the experimental assays. A Transwell containing BBB cells only, grown for 7 days, was used as control (CTRL). qRT-PCR of ABC transporter and tight junction (TJ) genes in BBB cells. Data are presented as means ± SD (n=3 independent experiments; each experimental point was performed in technical triplicates). *$p<0.05$, **$p<0.01$, ***$p<0.001$: AC/NS vs CTRL; °°$p<0.01$, °°°$p<0.001$: Nab-treated-AC vs AC, IL-6-treated NS vs NS.

Figure S10

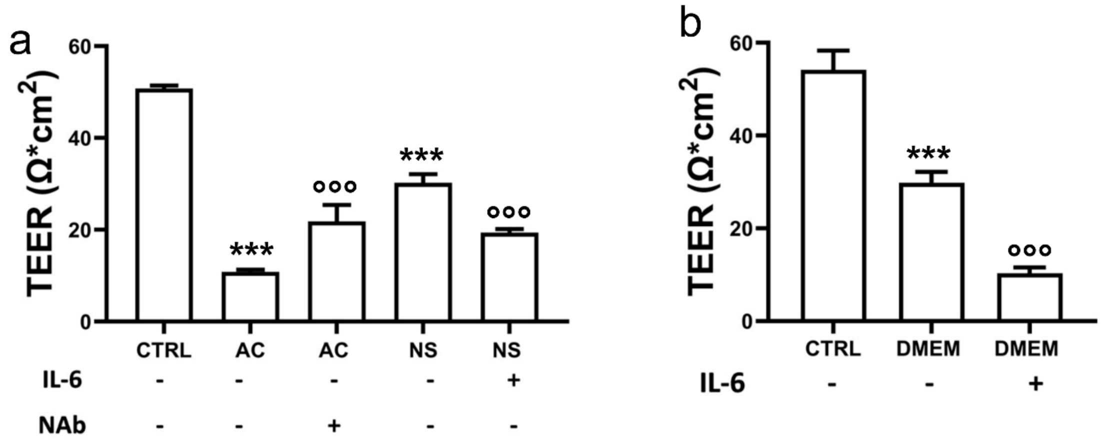


**Figure S10. TEER values of the blood-brain barrier in the presence of differentiated and stem cells-conditioned medium with different levels of IL-6. a.** Human blood-brain barrier (BBB) hCMEC/D3 cells were grown up to confluence in Transwell inserts. After 7 days, the conditioned medium derived from a 5-day culture of glioblastoma (GBM) cells of patient #2, as medium of differentiated/adherent cells (AC) or stem cell/neurospheres (NS), was added in the lower chamber for 72 h. Recombinant human IL-6 (200 pg/mL) was added in NS-conditioned medium, a neutralizing IL-6 antibody (Nab, 1/400) was added in AC-conditioned medium. A Transwell containing BBB cells only, grown for 7 days, was used as control (CTRL). Then TEER was measured on BBB. Data are presented as means ± SD (n=3 independent experiments; each experimental point was performed in technical duplicates). ***$p< 0.001$: AC/NS vs CTRL; °°°$p<0.001$: Nab-treated-AC vs AC, IL-6-treated NS vs NS. **b.** hCMEC/D3 cells were grown for 7 days up to confluence in Transwell insert, then incubated for 72 h with DMEM medium (used as internal control) and DMEM medium supplemented with recombinant human IL-6 (200 pg/mL) in the lower chamber. A Transwell containing BBB cells only, grown for 7 days, was used as baseline control (CTRL). TEER values of BBB. Data are presented as mean ± SD (n=3 independent experiments; each experimental point was performed in technical duplicates). ***$p< 0.005$: DMEM, DMEM+IL-6 vs CTRL; °°°$p<0.001$: DMEM+IL-6 vs DMEM.

# Figure S11

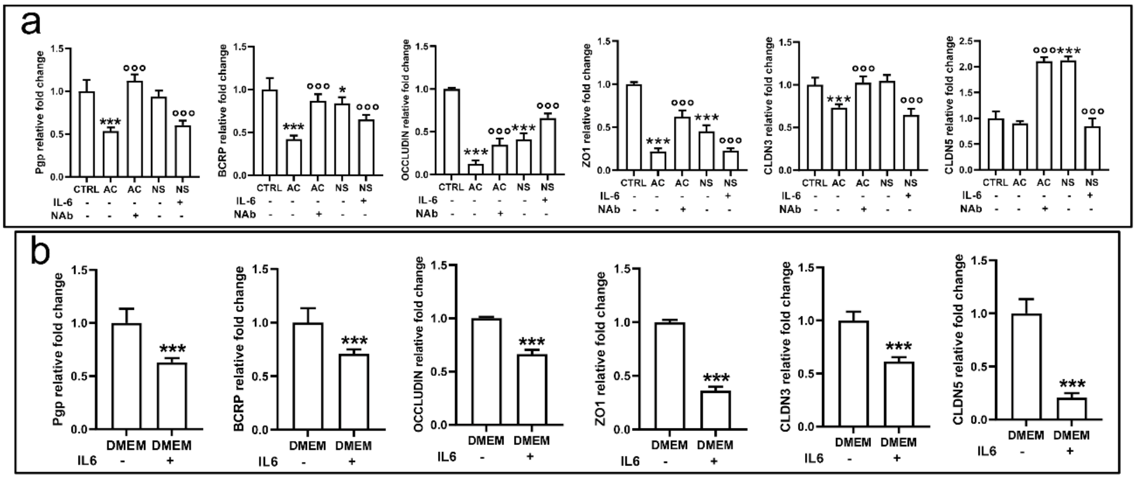


**Figure S11. Densitometric analysis of Figures 5d and 5e.** Densitometric analysis of immunoblot of Figure 5**d** (panel **a**) and Figure 5**e** (panel **b**). The ratio “band density of protein of interest/housekeeping protein” in CTRL cells was considered 1. Results were the ratio between “band density of protein of interest/housekeeping protein” in each experimental condition, expressed as fraction of the CTRL ratio. For panel **a**: *p<0.05, ***p< 0.001: AC/NS vs CTRL; °°°p<0.001: Nab-treated-AC vs AC, IL-6-treated NS vs NS; for panel **b**: ***p< 0.001: DMEM+IL-6 vs DMEM.

# Figure S12

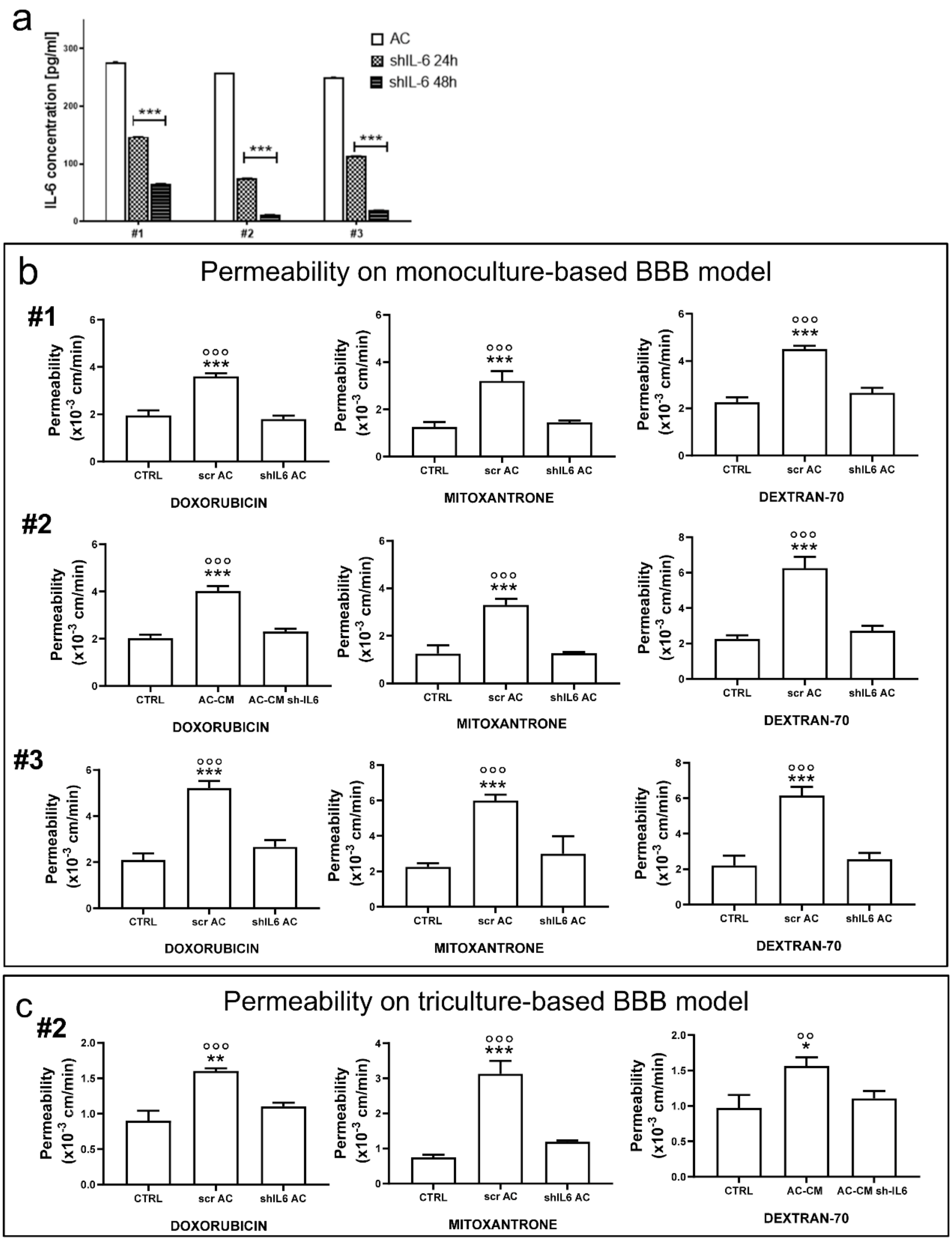


**Figure S12. The silencing of IL-6 in AC cells abrogates the effects of glioblastoma cells on BBB permeability.** AC from patient #1, #2, #3 were treated with a non-targeting scrambled shRNA vector (scr) or with an IL-6 silencing vector (shIL-6). **a.** ELISA detection of IL-6 in shIL-6 AC medium at 24 h and 48 h. Data are presented as mean ±

SD (n=3 independent experiments; each experimental point was performed in technical duplicates). ***p< 0.001: shIL-6 vs scr AC. For panels **b** and **c** the medium after 48 h silencing was used. **b.** Permeability assays in monoculture-based BBB model. Human blood-brain barrier (BBB) hCMEC/D3 cells were grown up to confluence in Transwell inserts. After 7 days, the conditioned medium derived from scr AC or shIL6 AC was added in the lower chamber for 72 h. A Transwell containing BBB cells only, grown for 7 days, was used as control (CTRL). 5 µM doxorubicin, 10 µM mitoxantrone or 2 µM dextran 70-fluorescein isothiocyanate were added in the Tranwell insert for 3 h. The compounds recovered from the medium of the lower chamber were measured fluorometrically. Data are presented as means ± SD (n=3 independent experiments; each experimental point was performed in technical duplicates). ***p< 0.001: scr AC vs CTRL; °°°p<0.001: shIL-6 vs CTRL. **c.** Permeability assays in triculture-based BBB model. Human blood-brain barrier (BBB) hCMEC/D3 cells with human astrocytes and pericytes were grown up to confluence in Transwell inserts, as reported in Supplementary Figure S6. After 7 days, the conditioned medium derived from scr AC or shIL6 AC #2 was added in the lower chamber for 72 h. A Transwell containing BBB cells only, grown for 7 days, was used as control (CTRL). 5 µM doxorubicin, 10 µM mitoxantrone or 2 µM dextran 70-fluorescein isothiocyanate were added in the Tranwell insert for 3 h. The compounds recovered from the medium of the lower chamber were measured fluorometrically. Data are presented as means ± SD (n=3 independent experiments; each experimental point was performed in technical duplicates). *p<0.05, **p<0.01, ***p< 0.001: scr AC vs CTRL; °°p<0.01, °°°p<0.001: shIL-6 vs CTRL.

# Figure S13

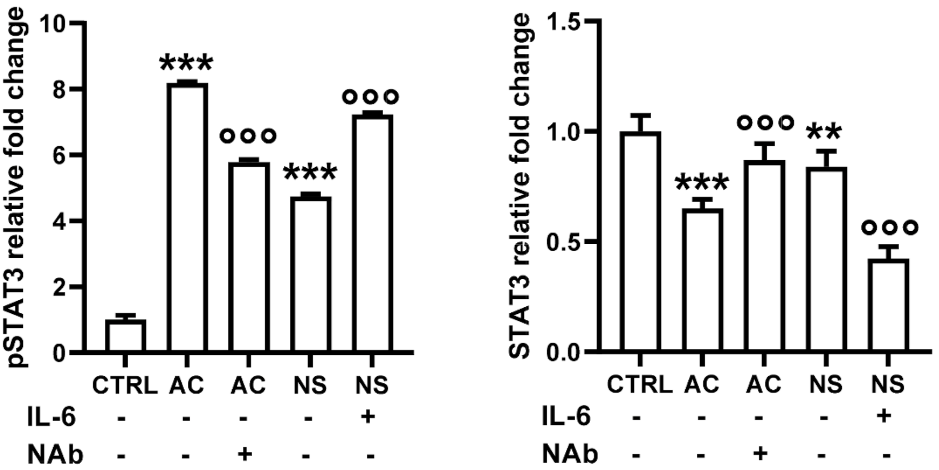


**Figure S13. Densitometric analysis of Figure 6b.** Densitometric analysis of immunoblot of Figure 6**b** (panel **c**). The ratio "band density of protein of interest/housekeeping protein" in CTRL cells was considered 1. Results were the ratio between "band density of protein of interest/housekeeping protein" in each experimental condition, expressed as fraction of the CTRL ratio. $^{**}p < 0.01$, $^{***}p < 0.001$: AC/NS vs CTRL; °°°$p<0.001$: Nab-treated-AC vs AC, IL-6-treated NS vs NS.

Figure S14

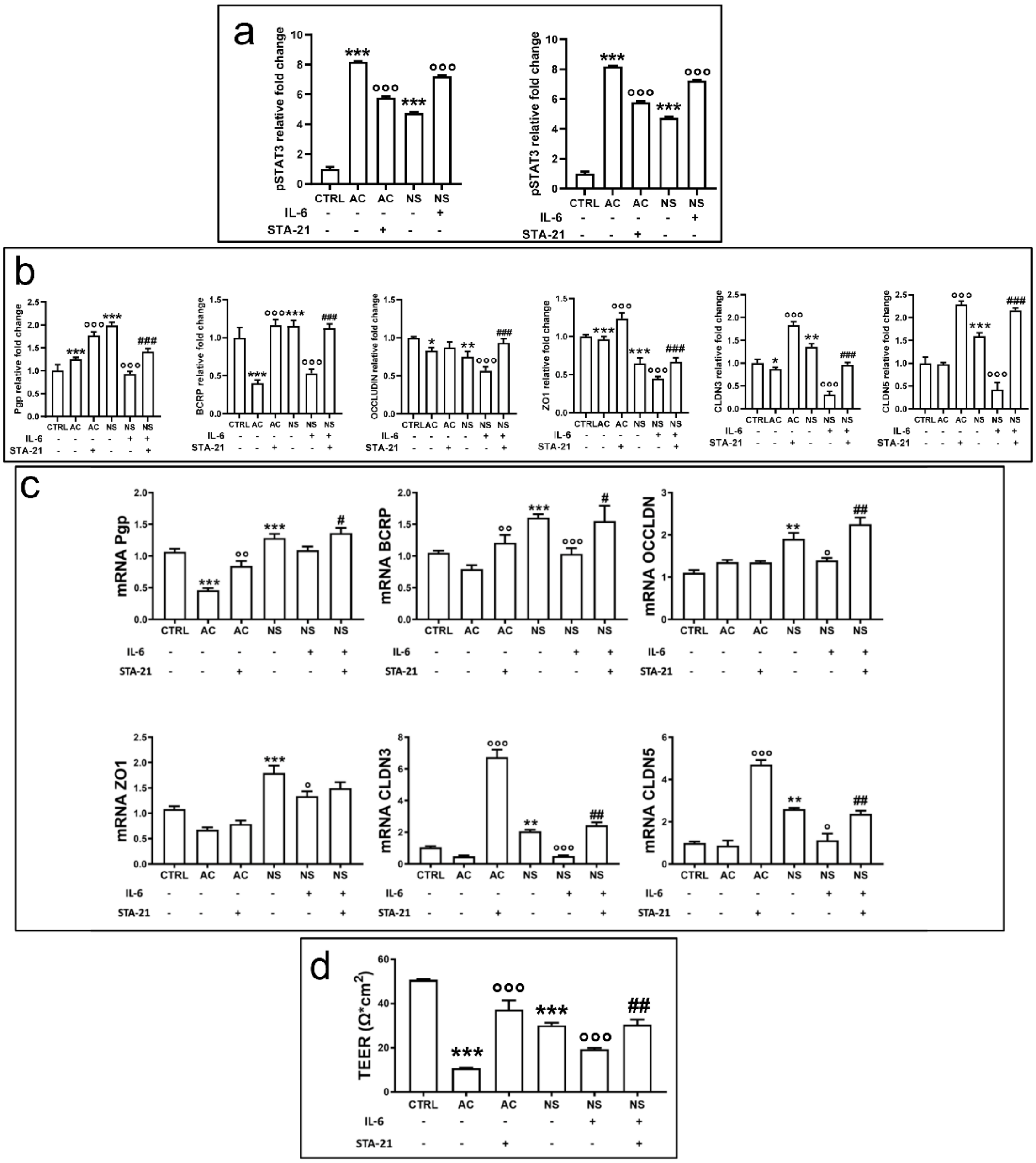


**Figure S14. The inhibition of STAT3 abrogates the effects of IL-6 on BBB permeability.** Human blood-brain barrier (BBB) hCMEC/D3 cells were grown up to confluence in Transwell inserts. After 7 days, the conditioned medium derived from a 5-day culture of glioblastoma (GBM) cells of #2 patient, in the form of medium of differentiated/adherent cells (AC) or stem cell/neurospheres (NS), was added in the lower chamber for 72 h. BBB was exposed to non-conditioned medium (CTRL), AC- or NS-conditioned medium, NS-conditioned medium containing human recombinant IL-6 (200 pg/mL). When indicated the inhibitor of STAT3, STA-21 (30 µM) was co-incubated. **a-b**. Densitometric analysis of immunoblot Figure 6**c** (panel **a**) and Figure 6**d** (panel **b**).

The relative band density of protein of interest/housekeeping protein in CTRL cells was considered 1. Results were the ratio between "band density of protein of interest/housekeeping protein" in each experimental condition, expressed as fraction of the CTRL ratio. For panel **a**: ***p< 0.001: AC/NS vs CTRL; °°°p<0.001: Nab-treated-AC vs AC, IL-6-treated NS vs NS. For panel **b**: *p<0.05, **p<0.01, ***p< 0.001: AC/NS vs CTRL; °°°p<0.001: STA21-treated-AC vs AC, IL-6-treated NS vs NS; $^{###}$p<0.001: STA-21+IL-6 treated NS vs IL-6 treated NS. **c**. qRT-PCR of ABC transporter and tight junction (TJ) genes in BBB cells. Data are presented as means ± SD (n=3 independent experiments; each experimental point was performed in technical triplicates). **p<0.01, ***p< 0.001: AC/NS vs CTRL; °p<0.05, °°p<0.01, °°°p<0.001: STA21-treated-AC vs AC, IL-6-treated NS vs NS; $^{#}$p<0.05, $^{##}$p<0.01; STA-21+IL-6 treated NS vs IL-6 treated NS. **d**. TEER values of cells incubated as in Figure 6**e**. Data are presented as mean ± SD (n=3 independent experiments; each experimental point was performed in technical duplicates). ***p<0.001: AC/NS vs CTRL; °°°p<0.001: STA21-treated-AC vs AC, IL-6-treated NS vs NS; $^{##}$p<0.01: STA-21+IL-6 treated NS vs IL-6 treated NS.

# Figure S15

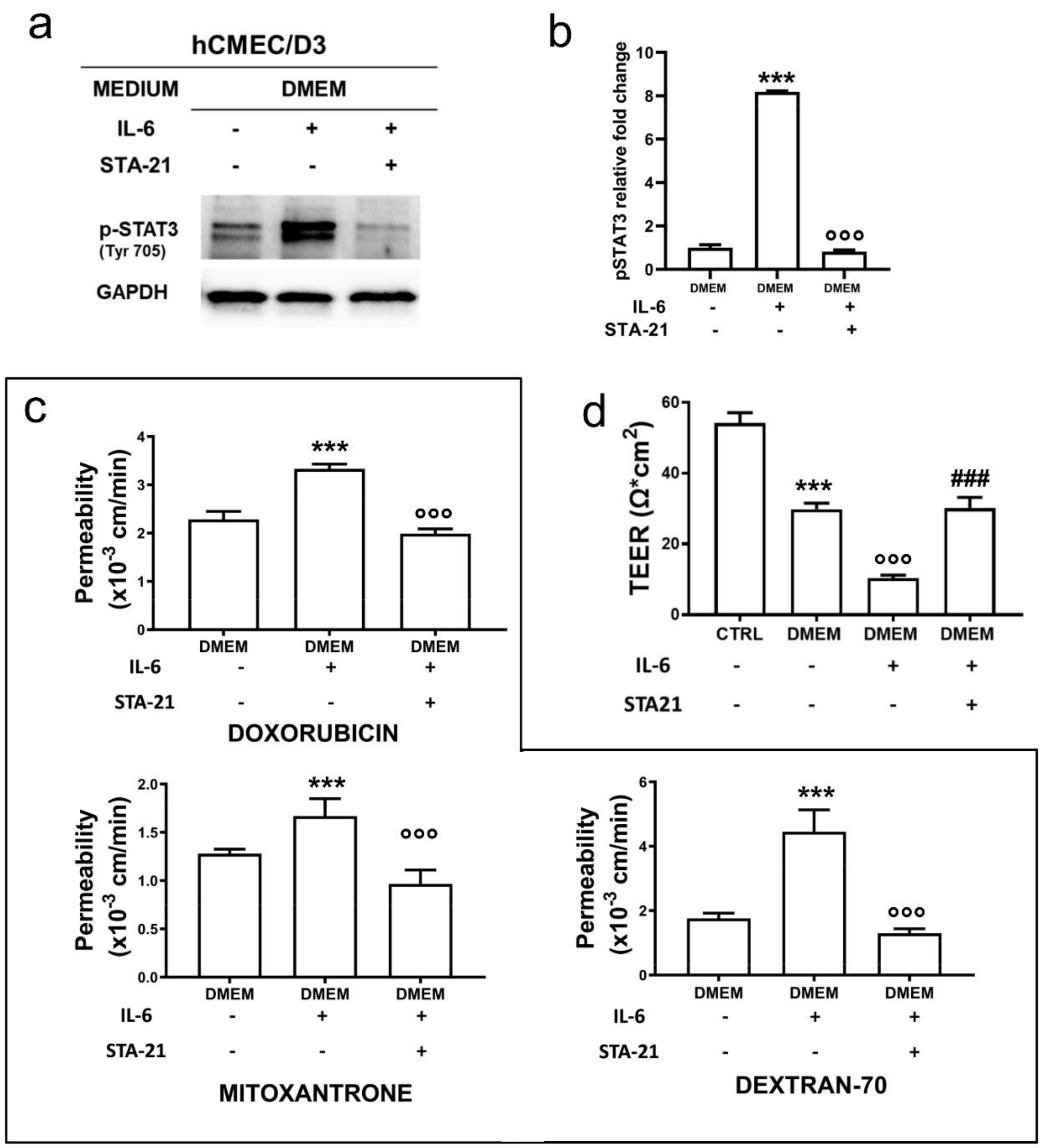


**Figure S15. STA-21 abrogates the effect of IL-6.** Blood-brain barrier (BBB) hCMEC/D3 cells were grown for 7 days up to confluence in Transwell insert, then incubated for 72 h with DMEM medium (used as internal control) and DMEM medium supplemented with recombinant human IL-6 (200 pg/mL) in the lower chamber. When indicated, the inhibitor of STAT3, STA-21 (30 µM) was co-incubated. **a**. Immunoblotting of STAT3 and phospho(Tyr705)STAT3. The expression of GAPDH was used as control of equal protein loading. The figure is representative of one out of three experiments with similar results. **b**. Densitometric analysis of panel **a**. The relative band density of protein of interest/housekeeping protein in DMEM (IL-6-, STA21-) cells was considered 1. Results were the ratio between "band density of protein of interest/housekeeping protein"

in each experimental condition, expressed as fractio of the DMEM ratio. *** p<0.001: DMEM+IL6 vs DMEM; °°°p<0.001: DMEM+IL-6+STA-21 vs DMEM+IL-6. **c**. Permeability assay on BBB cells treated as in **a**, measured fluorometrically. Data are presented as means ± SD (n=3 independent experiments; each experimental point was performed in technical duplicates). ***p<0.001: DMEM+IL6 vs DMEM; °°°p<0.001: DMEM+IL-6+STA-21 vs DMEM+IL-6. **d**. TEER values of BBB cells treated as in **a**. A Transwell containing BBB cells only, grown for 7 days, was used as control (CTRL). Data are presented as means ± SD (n=3 independent experiments; each experimental point was performed in technical duplicates). ***p<0.001: DMEM vs CTRL; °°°p<0.001: DMEM+IL-6 vs DMEM; $^{###}$p<0.001: DMEM+IL-6+STA-21 vs DMEM+IL-6.

Figure S16

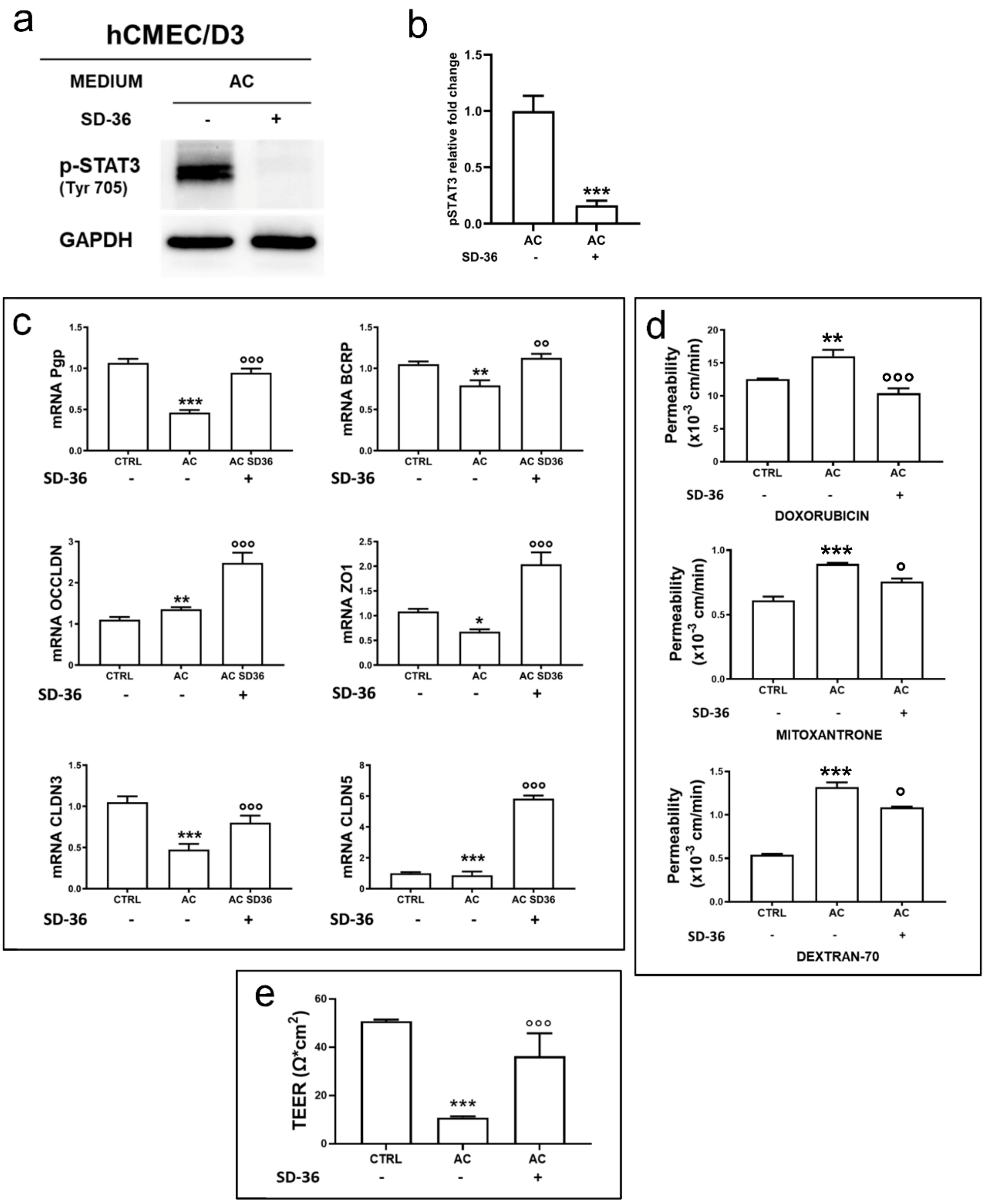


**Figure S16. STAT3-targeting PROTAC recapitulates the effects of inhibiting IL-6/STAT3 in blood-brain barrier cells exposed to the medium of differentiated cells.** Human (BBB) hCMEC/D3 blood-brain barrier cells were grown up to confluence in Transwell inserts. After 7 days, the conditioned medium derived from a 5-day culture of glioblastoma (GBM) cells of patient #2, in the form of medium of differentiated/adherent cells (AC), was added in the lower chamber for 72 h, alone or in the presence of the STAT3 PROTAC SD-36 (1 µM). **a**. Immunoblotting of phospho(Tyr705)STAT3. The

expression of GAPDH was used as control of equal protein loading. The figure is representative of one out of three experiments with similar results. **b**. Densitometric analysis of panel **a**. ***$p<0.05$: SD-36+AC vs AC. **c**. qRT-PCR of ABC transporter and tight junction (TJ) genes in BBB cells, in cultures as in **a**. A Transwell containing BBB cells only, grown for 7 days, was used as control (CTRL). Data are presented as means ± SD (n=3 independent experiments; each experimental point was performed in technical triplicates). *$p<0.05$, **$p<0.01$, ***$p<0.001$: AC vs CTRL; °°$p<0.01$, °°°$p<0.001$: SD-36+AC vs AC. **d**. Permeability assay on BBB cells treated as **c**, measured fluorometrically. Data are presented as means ± SD (n=3 independent experiments; each experimental point was performed in technical duplicates). **$p<0.01$, ***$p<0.001$: AC vs CTRL; °$p<0.05$, °°°$p<0.001$: SD-36+AC vs AC. **e**. TEER values in the same conditions of **c**. Data are presented as means ± SD (n=3 independent experiments; each experimental point was performed in technical duplicates). ***$p<0.001$: AC vs CTRL; °°°$p<0.001$: AC SD-36 vs AC.
